# Supplementary material for: Automated navigation of condensate phase behavior with active machine learning
Source: Nat Commun. 2025 Oct 30;16:9598. doi: 10.1038/s41467-025-64617-2 (PMC12575655; doi:10.1038/s41467-025-64617-2)
Supplement: Supplementary file 1 — Supplementary Information [file 41467_2025_64617_MOESM1_ESM.pdf]

Supplementary Information

Automated navigation of condensate phase behavior with active machine learning

Y.H.A. Leurs[+], W. van den Hout[+], A. Gardin[+], J.L.J. van Dongen, Andoni Rodriguez-Abetxuko, Nadia A. Erkamp, J.C.M. van Hest*, F. Grisoni*, L. Brunsveld*

Institute for Complex Molecular Systems (ICMS), Department of Biomedical Engineering, Eindhoven University of Technology, PO Box 513, 5600 MB Eindhoven, The Netherlands.

[+]These authors contributed equally.

*j.c.m.v.hest@tue.nl, f.grisoni@tue.nl, l.brunsveld@tue.nl

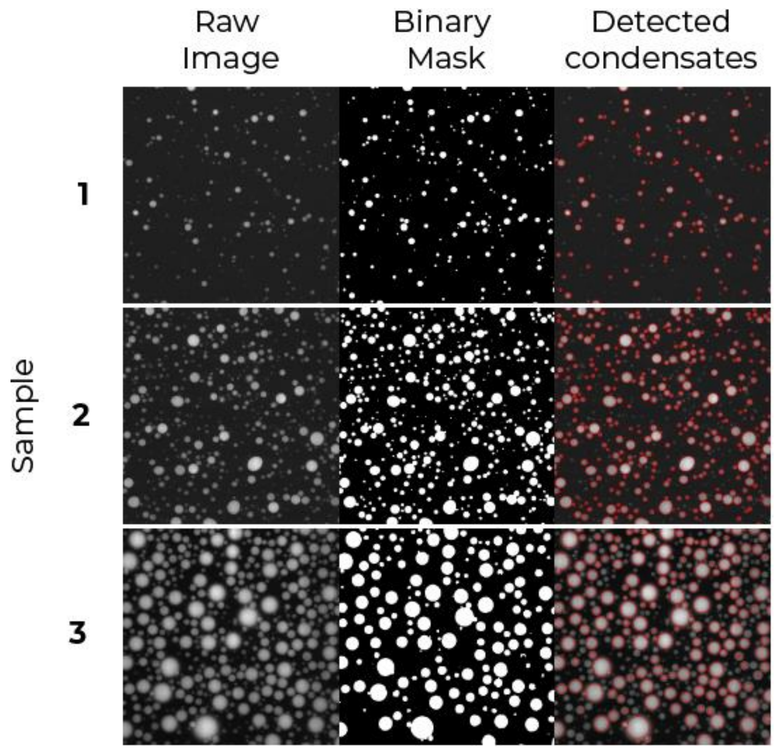

Supplementary Figure 1: Representative confocal micrographs, segmentation, and particle detection of varying condensate forming conditions of poly-*L*-(lysine)$_{100}$ and poly-*L*-(aspartic acid)$_{200}$. From left to right: the raw confocal image, the corresponding binary mask obtained by Yen-thresholding, and an overlay highlighting detected coacervates above the 500 pixel threshold (in red). All samples contain 150 mM NaCl, with increasing concentrations of aspartic acid (2.5, 4.1, 8.1 mM), lysine (3.7, 6.0, 7.5 mM), and imaging depths of 1, 3, and 7 μm for samples 1–3, respectively. These images depict the segmentation and particle detection methods employed in the automated image analysis throughout all experiments.

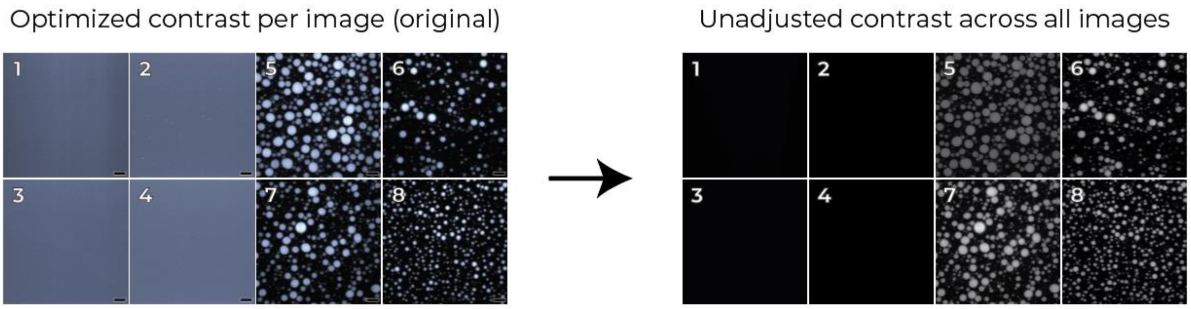

Supplementary Figure 2: Effect of contrast scaling on the interpretability of confocal images. *Left:* Images with individually optimized contrast to enhance visibility and distinguish negative (1–4) from positive (5–8) samples. This approach was used in the main manuscript to maximize contrast in each image. *Right:* Same images with uniform global contrast. While preserving raw intensity values, this renders panes 1–4 nearly black and visually uninformative. Additionally, pane 5 appears dimmer due to reduced dye accumulation in these droplets.

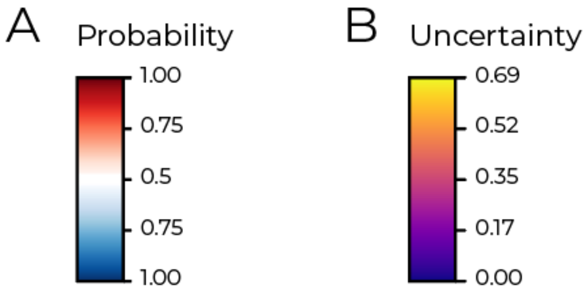

Supplementary Figure 3: Scalebars. (**A**) Probability and (**B**) uncertainty scalebars that are consistently applied across the Supplementary Figures in this document. Probability ranges from blue (phase separation) to red (no phase separation), while uncertainty ranges from 0 (low entropy) to 0.69 (high entropy).

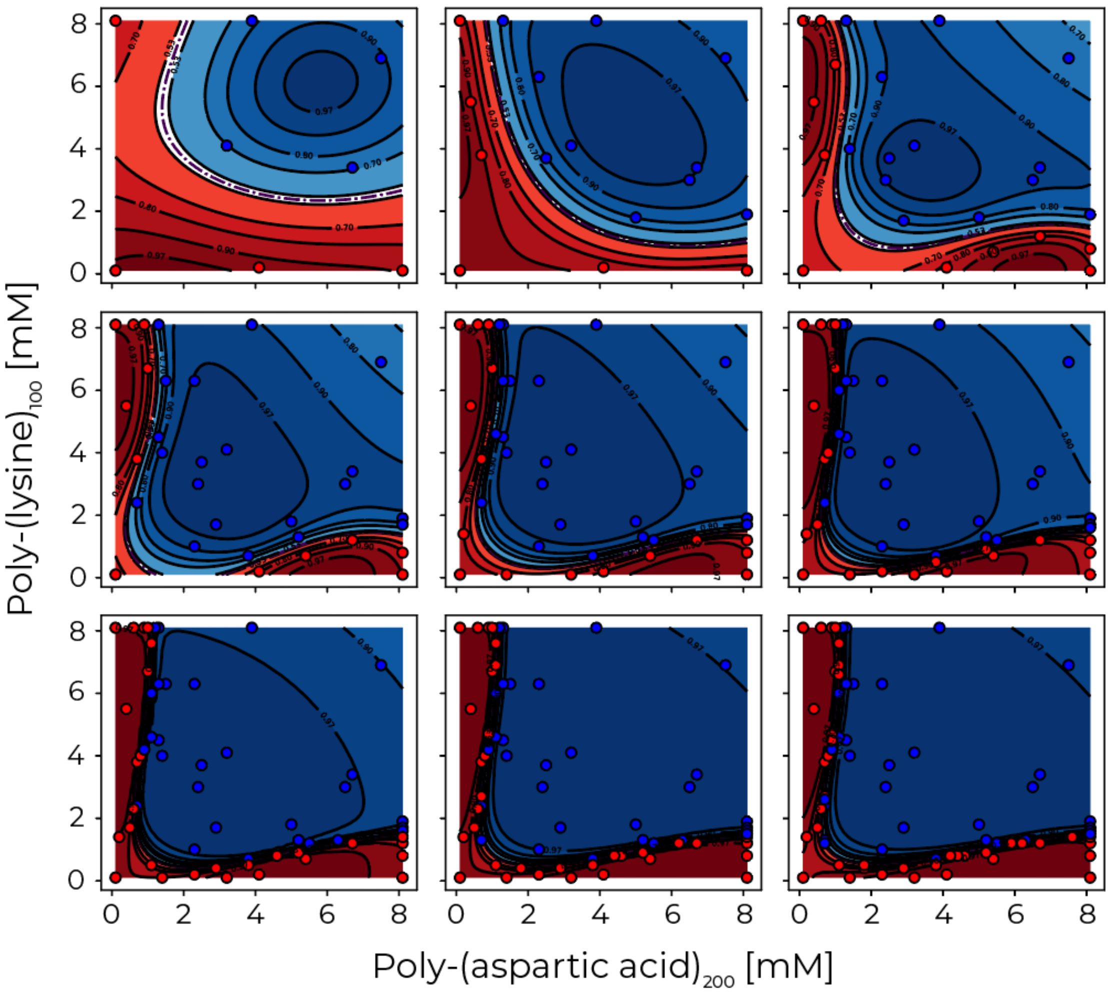

Supplementary Figure 4: Convergence of the poly-L-(lysine)$_{100}$ and poly-L-(aspartic acid)$_{200}$ condensate phase diagram. This figure shows 72 datapoints acquired over 9 cycles. Blue points represent phase separation, while red points indicate no phase separation. Axes represent monomer concentrations of each component (mM). The corresponding entropy map is provided in Supplementary Figure 5. The background surface represents the model's predictions, with the scalebar provided in Supplementary Figure 3A.This overview corresponds to the data presented in the main text, Figure 2.

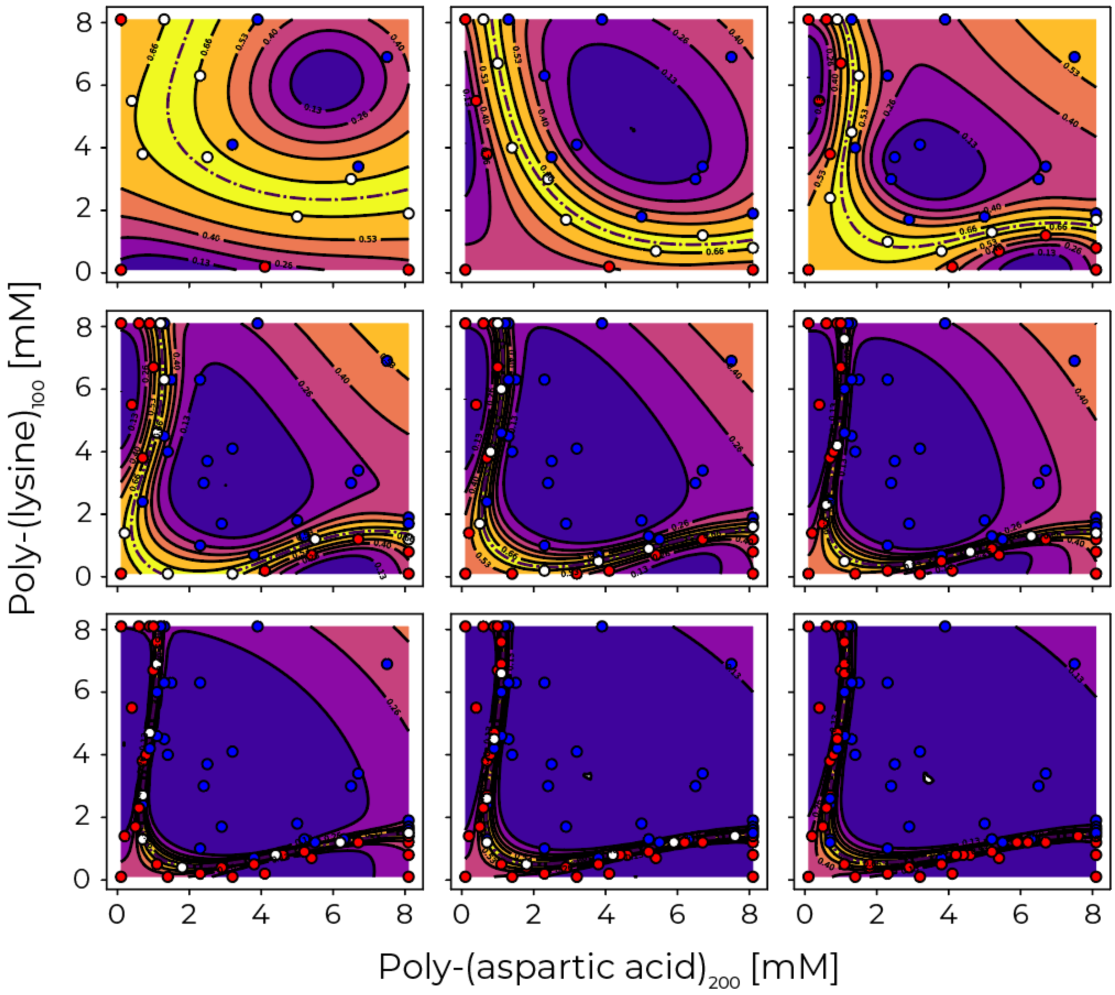

Supplementary Figure 5: Entropy map of the poly-L-(lysine)$_{100}$ and poly-L-(aspartic acid)$_{200}$ condensate phase diagram. This figure shows the entropy maps for the phase diagrams in Supplementary Figure 4. Blue points represent phase separation, red points indicate no phase separation, and white points indicate the newly requested datapoints. This figure highlights the progression of phase mapping using farthest point sampling guided by entropy values with one-decimal precision. Axes represent monomer concentrations of each component (mM). The background surface represents the model's uncertainty, with the scalebar provided in Supplementary Figure 3B.

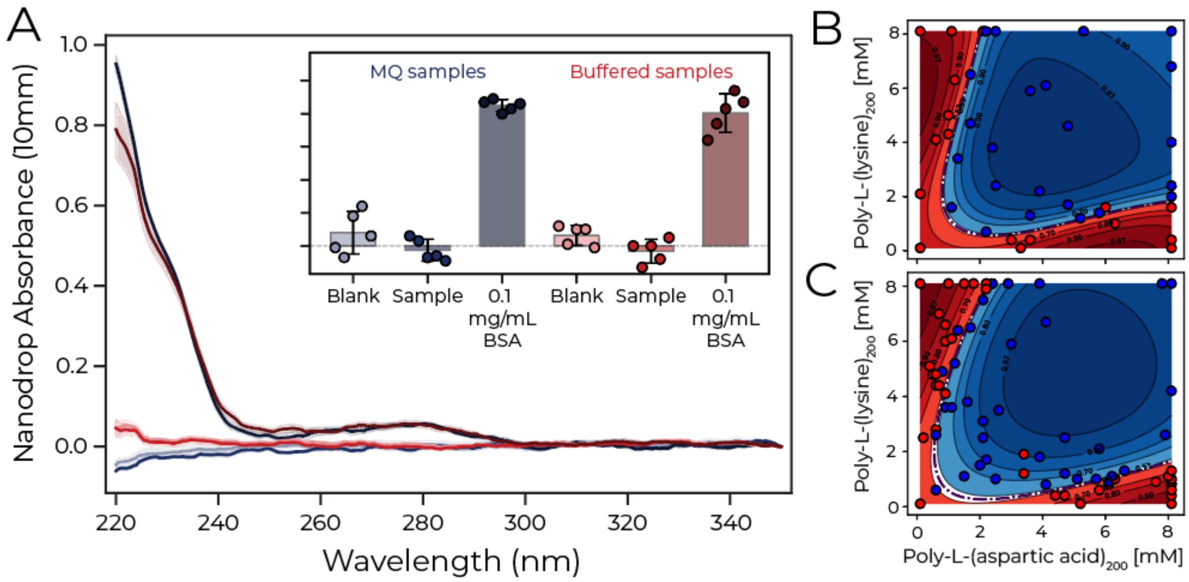

Supplementary Figure 6: Assessment of BSA carryover effects on phase behavior. (A) Nanodrop absorbance at 280 nm for solutions incubated in BSA-coated wells for 15 minutes with either ultrapure water (left) or experimental buffer (right), compared to a blank and a 0.1 mg/mL BSA reference. No absorbance was detected above background, indicating negligible BSA desorption. **(B–C)** Phase diagrams of poly-*L*-(lysine)$_{100}$ and poly-*L*-(aspartic acid)$_{200}$ acquired without (**B**) or with (**C**) 0.001 mg/mL BSA supplemented in the bulk solution. This concentration reflects a conservative upper bound assuming complete BSA desorption from the plate. No differences in phase behavior were observed.

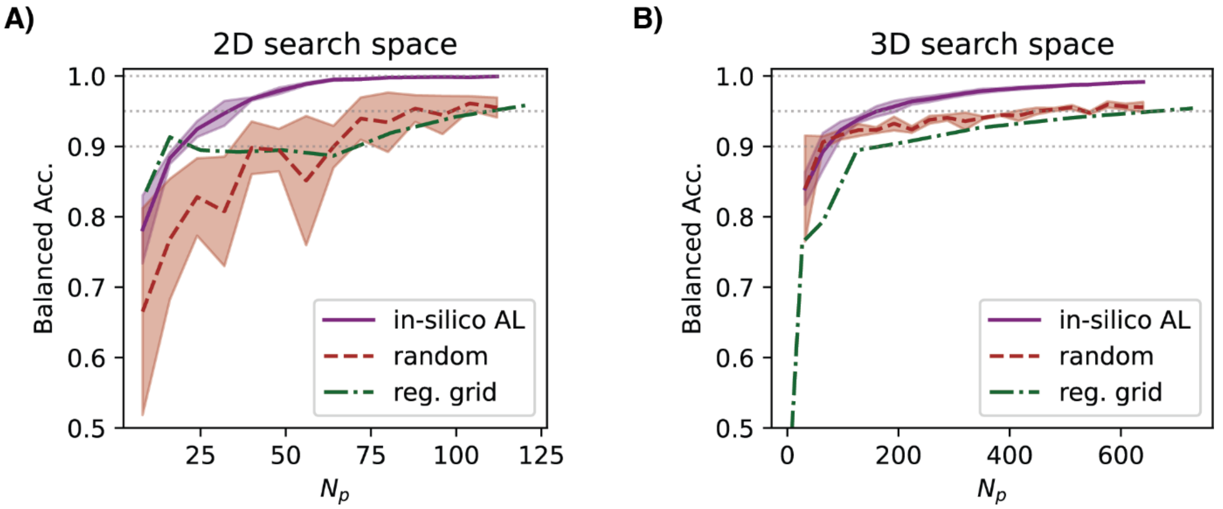

Supplementary Figure 7: Comparison of Active Learning (AL) and non-Machine Learning (ML) sampling methods for phase diagram screening. A) Results for the 2D system (poly-*L*-(lysine)$_{100}$ / poly-*L*-(aspartic acid)$_{200}$; 6561 points), showing the balanced accuracy between the predicted phase diagrams and the collected ground truth for three sampling strategies: in-silico AL (solid purple line), random sampling (dashed brown line), and regular grid sampling (dash-dotted dark green line). In each iteration, selected data points were used to retrain a Gaussian Process Classifier, with AL following the same iterative procedure as in the experimental workflow. Replicates: random (n = 5), AL (n = 3), grid (deterministic). **B)** Results for the 3D system (poly-*L*-(lysine)$_{100}$ / poly-*L*-(aspartic acid)$_{200}$ / NaCl; 531,441 points), using the same evaluation as (**A**). Shaded regions indicate the standard deviation computed over multiple replicates (n=5 random sampling, n=3 for AL; grid sampling is deterministic).

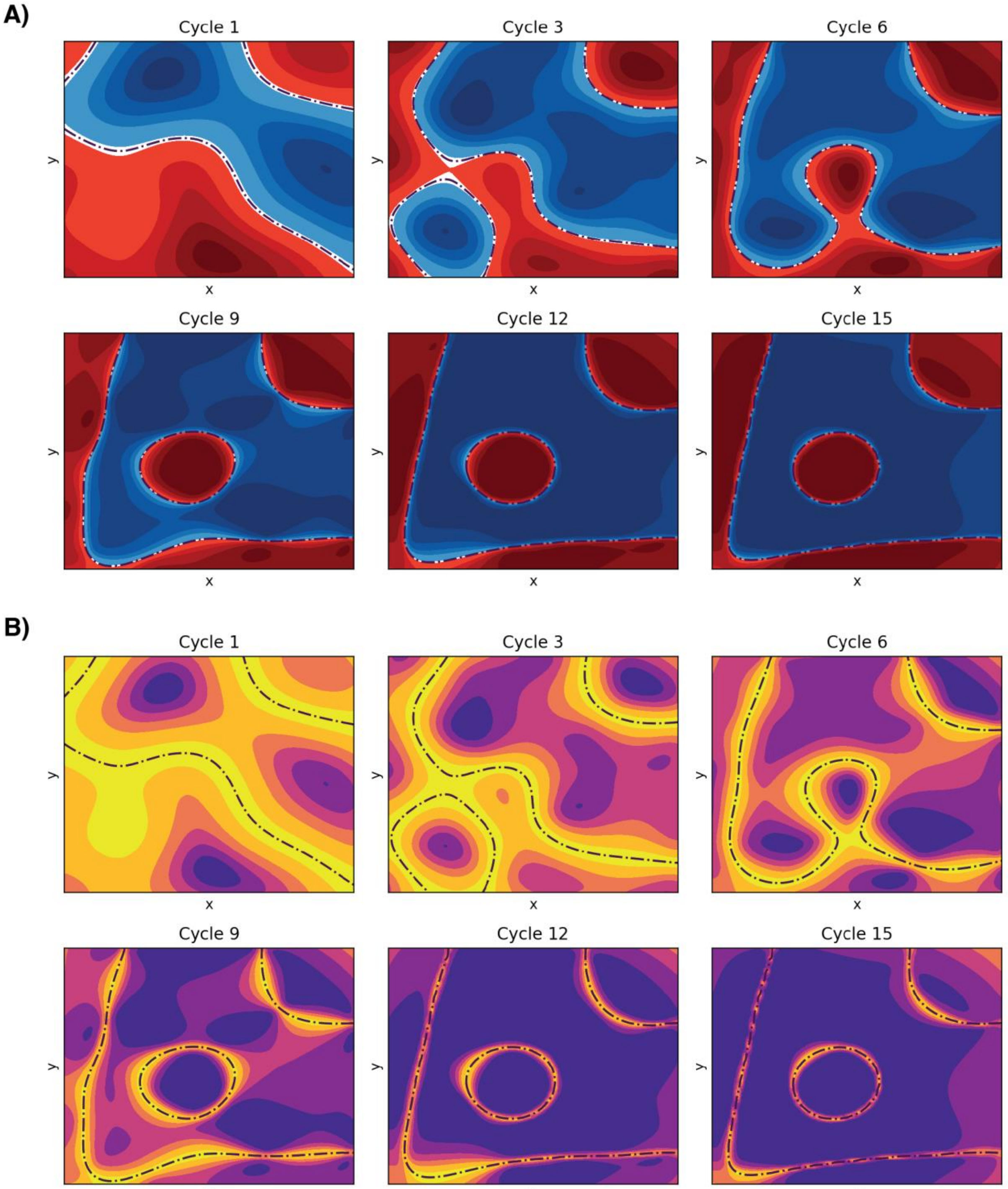

Supplementary Figure 8: *In-silico* validation of the active machine learning framework for resolving complex phase landscapes. To evaluate the platform's ability to detect unexpected or highly localized phase boundaries, we constructed an artificial phase diagram with increased complexity. (**A**) The evolving probability landscape over 15 cycles demonstrates how the model progressively detects and outlines distinct phase regions separated from the main boundary. (**B**) The corresponding uncertainty maps illustrate a consistent reduction in prediction entropy across cycles, reflecting improved model certainty. Color scales for both panels are provided in Supplementary Figure 3. These results demonstrate that the active machine learning framework remains effective even when challenged with more complex and fragmented phase landscapes, successfully resolving subtle and spatially separated features.

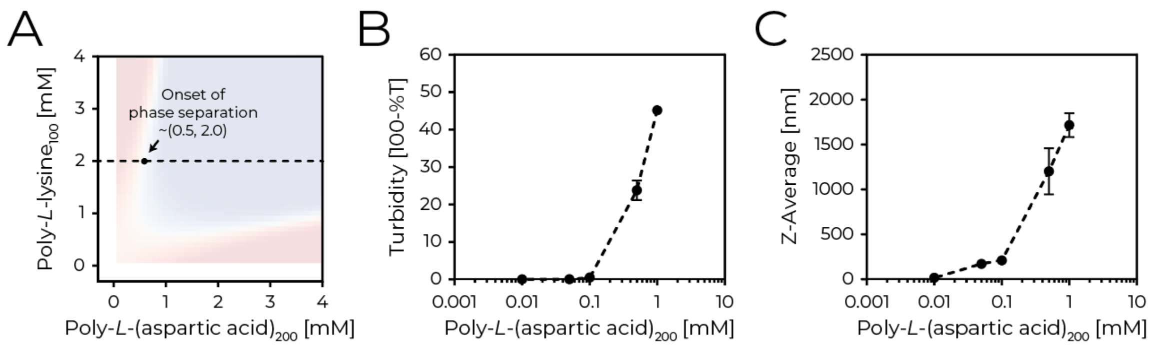

Supplementary Figure 9: Characterization of poly-*L*-(lysine)$_{100}$ / poly-*L*-(aspartic acid)$_{200}$ formulations near the predicted phase boundary via turbidity and dynamic light scattering (DLS). (**A**) Zoom-in of the analyzed region within the phase diagram. The background gradient corresponds to the phase probability of the "ground-truth" phase diagram used in the main text (Figure 4). Experimental formulations were prepared along the dotted line at a fixed 2.0 mM of poly-*L*-(lysine)$_{100}$ with varying of poly-*L*-(aspartic acid)$_{200}$ concentrations (0.01–1.0 mM) in 50 mM HEPES (pH 7.4) with 150 mM NaCl. The approximate onset of phase separation along this trajectory was identified by the automated analysis workflow at around 2.0 mM lysine and 0.5 mM aspartic acid (black dot). (**B**) Turbidity (λ = 600 nm) as a function of poly-*L*-(aspartic acid)$_{200}$ concentration. Values remain low at concentrations (<0.1 mM) and increase sharply above 0.5 mM, indicating the onset of phase separation. (**C**) DLS-derived Z-average hydrodynamic diameters for the same formulations as in (**B**). Nanometer-scale particles (~170–210 nm) are detected between 0.05 and 0.1 mM poly-*L*-(aspartic acid)$_{200}$, while micrometer-scale droplets are observed above 0.5 mM. All measurements were performed 15 minutes after sample preparation, consistent with the timing used throughout the automated experimental pipeline. Data represent mean ± SD (n = 3).

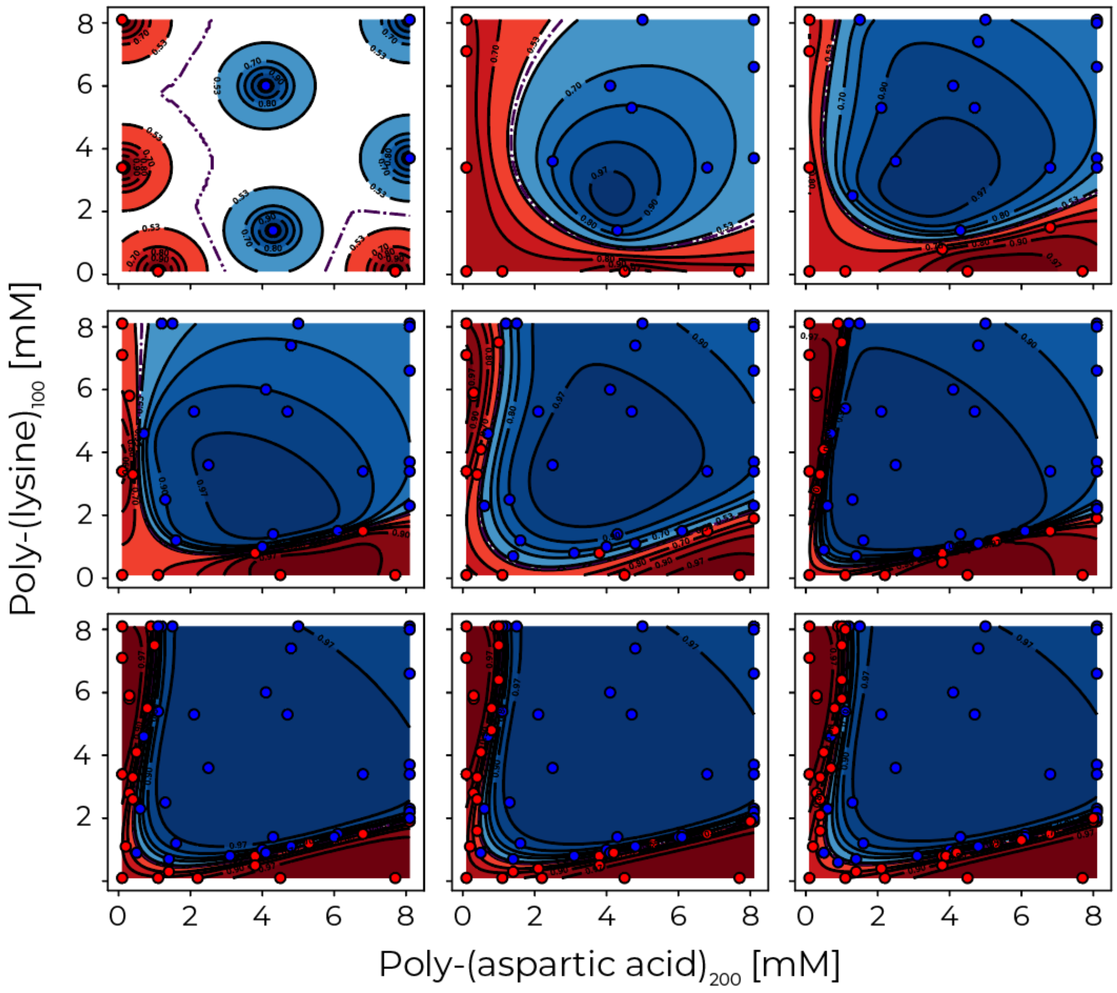

Supplementary Figure 10: Convergence of the poly-L-(lysine)$_{100}$ and poly-L-(aspartic acid)$_{200}$ condensate phase diagram (Run 2). This figure shows a replicate experiment with 72 datapoints acquired over 9 cycles. The same conditions were used as in Supplementary Figure 4, and a selection of the data is shown in the main text in Figure 3 (Run 2). Blue points represent phase separation, while red points indicate no phase separation. Axes represent monomer concentrations of each component (mM). The corresponding entropy map is provided in Supplementary Figure 11. The background surface represents the model's predictions, with the scalebar provided in Supplementary Figure 3A.

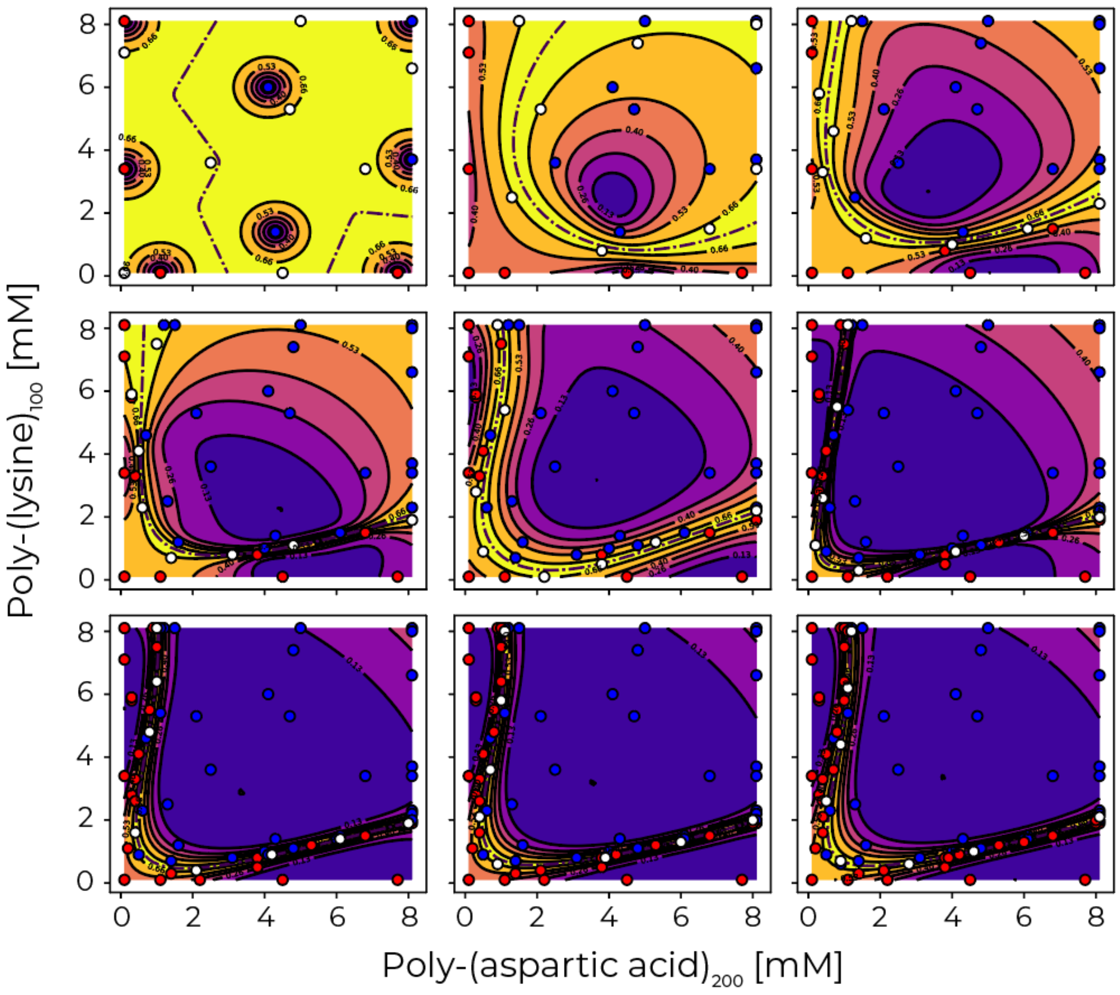

Supplementary Figure 11: Entropy map of the poly-L-(lysine)$_{100}$ and poly-L-(aspartic acid)$_{200}$ condensate phase diagram (Run 2). This figure shows the entropy maps for the replicate experiment in Supplementary Figure 10. Blue points represent phase separation, red points indicate no phase separation, and white points indicate the newly requested datapoints. Axes represent monomer concentrations of each component (mM). The background surface represents the model's uncertainty, with the scalebar provided in Supplementary Figure 3B.

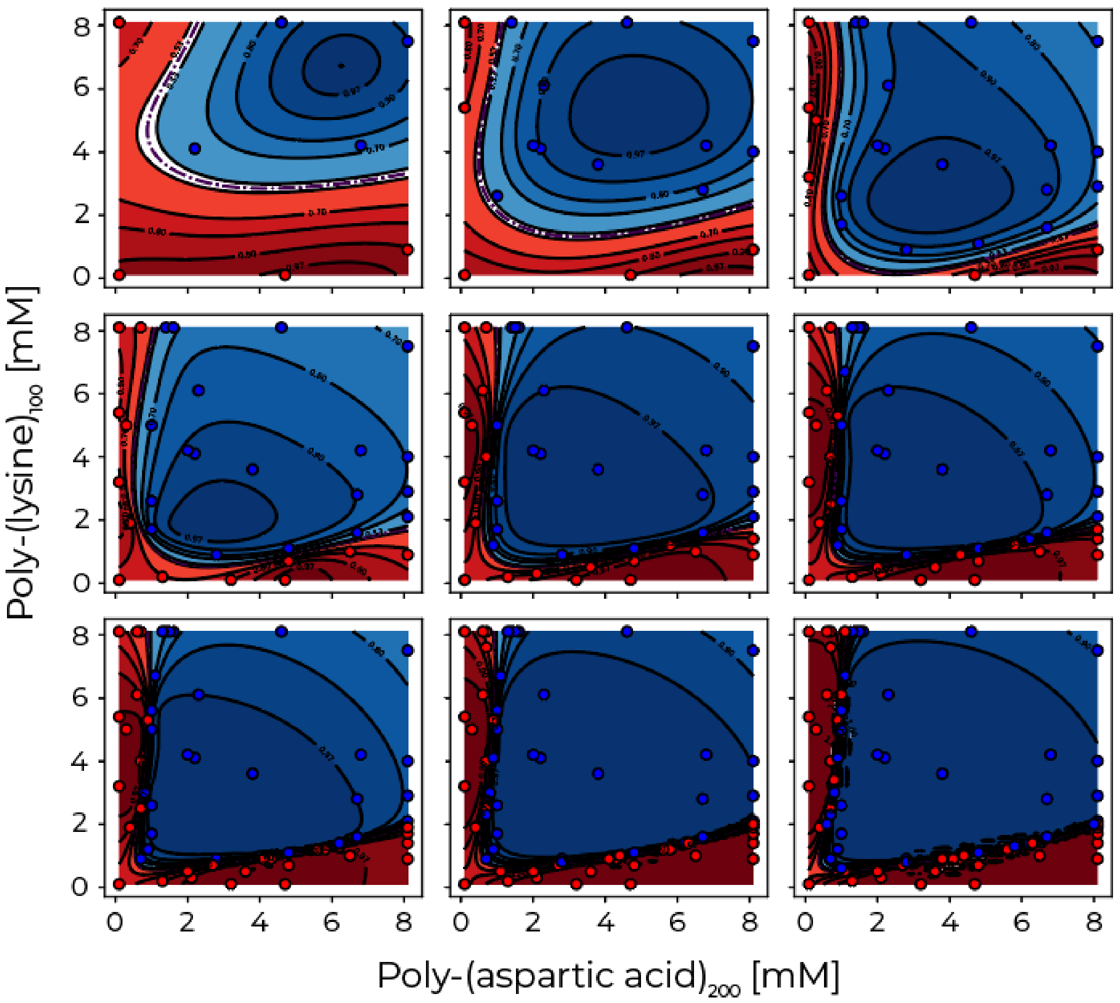

Supplementary Figure 12: Convergence of the poly-L-(lysine)$_{100}$ and poly-L-(aspartic acid)$_{200}$ condensate phase diagram (Run 3). This figure shows a replicate experiment with 72 datapoints acquired over 9 cycles. The same conditions were used as in Supplementary Figure 4, and a selection of the data is shown in the main text in Figure 3 (Run 3). Blue points represent phase separation, while red points indicate no phase separation. Axes represent monomer concentrations of each component (mM). The corresponding entropy map is provided in Supplementary Figure 13. The background surface represents the model's predictions, with the scalebar provided in Supplementary Figure 3A.

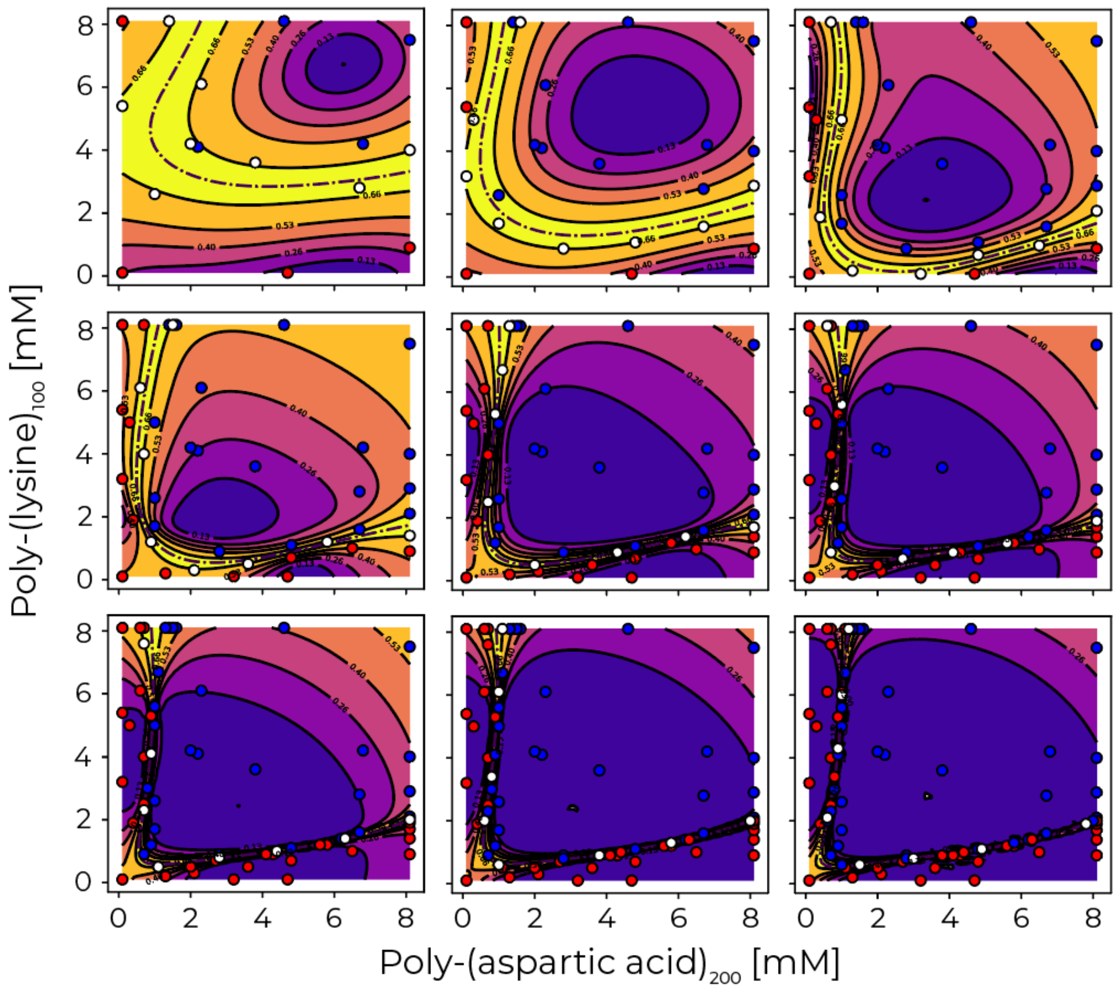

Supplementary Figure 13: Entropy map of the poly-L-(lysine)$_{100}$ and poly-L-(aspartic acid)$_{200}$ condensate phase diagram (Run 3). This figure shows the entropy maps for the replicate experiment in Supplementary Figure 12. Blue points represent phase separation, red points indicate no phase separation, and white points indicate the newly requested datapoints. Axes represent monomer concentrations of each component (mM). The background surface represents the model's uncertainty, with the scalebar provided in Supplementary Figure 3B.

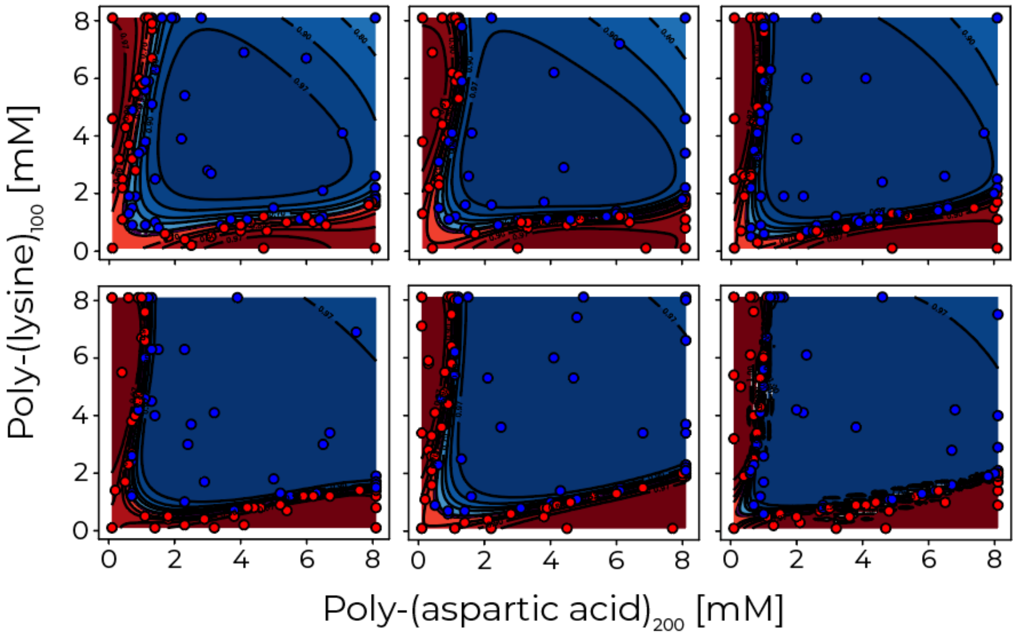

Supplementary Figure 14: All datapoints used to construct the "ground truth" phase diagram for poly-L-(lysine)$_{100}$ and poly-L-(aspartic acid)$_{200}$. The upper panel shows data from three optimization experiments (Supplementary Figures 15-20). The lower panel presents data from the three independent experimental replicates discussed in the main text, Figure 3, and Supplementary Figures 4-5 and Supplementary Figures 10-13. A total of 480 datapoints are included across six experiments, plotted against monomer concentrations of each component. Blue points represent phase separation, while red points indicate no phase separation. The surface represents the model's predictions, with the scalebar provided in Supplementary Figure 3A.

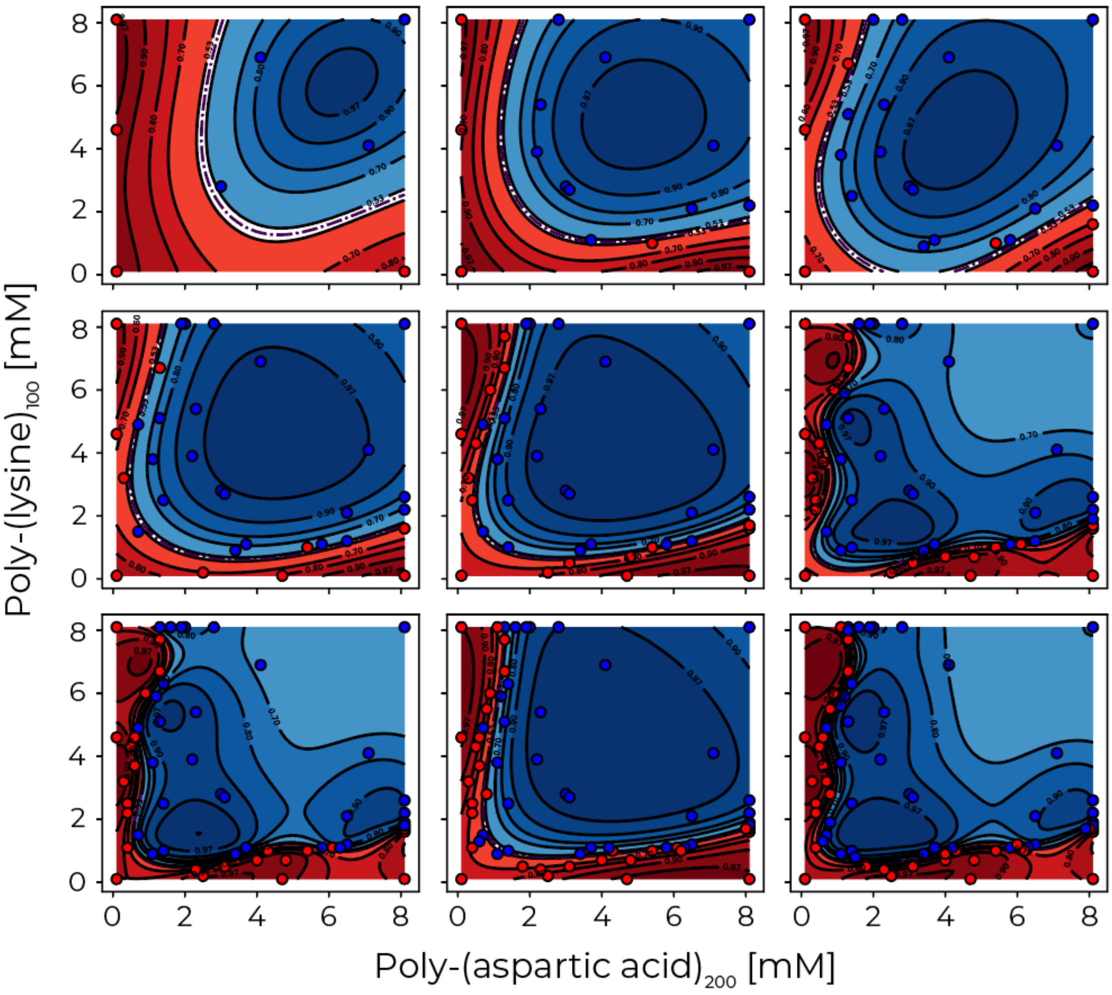

Supplementary Figure 15: Convergence of the poly-L-(lysine)$_{100}$ and poly-L-(aspartic acid)$_{200}$ condensate phase diagram (optimization experiment, Run 1). This figure shows Run 1 of three optimization experiments where we used farthest point sampling guided by entropy values with two-decimal precision, different from the one-decimal precision used in main text Figure 3 and Supplementary Figures 4-5 and Supplementary Figures 10-13. For comparison, the first 72 datapoints acquired over 9 cycles are shown, highlighting the effect of using suboptimal sampling conditions. Blue points represent phase separation, while red points indicate no phase separation. Replicates using the same conditions are shown in Supplementary Figures 17-20. The corresponding entropy map is provided in Supplementary Figure 16. The background surface represents the model's predictions, with the scalebar provided in Supplementary Figure 3A. Axes represent monomer concentrations of each component (mM).

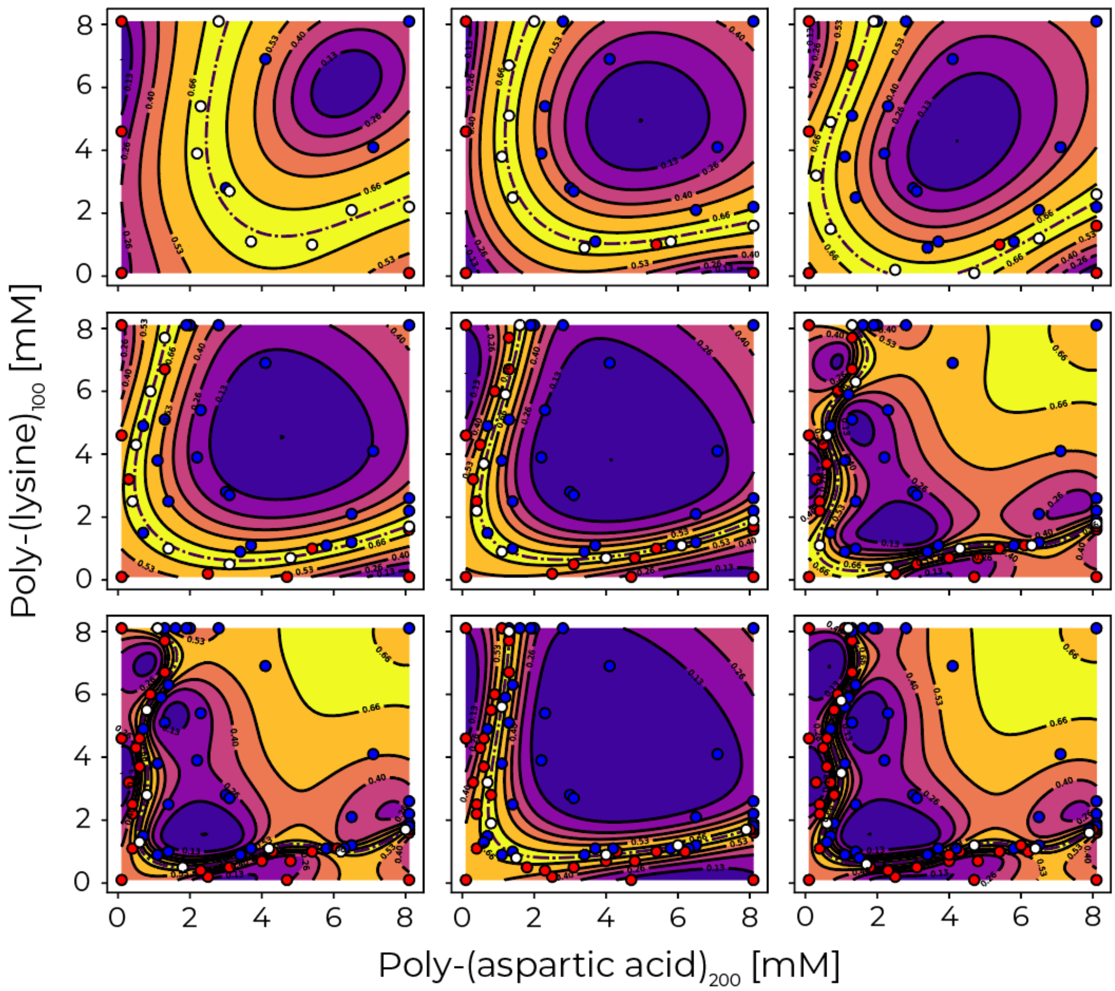

Supplementary Figure 16: Entropy map of the poly-L-(lysine)$_{100}$ and poly-L-(aspartic acid)$_{200}$ condensate phase diagram (optimization experiment, Run 1). This figure shows the entropy maps for the phase diagrams in Supplementary Figure 15. Blue points represent phase separation, red points indicate no phase separation, and white points indicate the newly requested datapoints. Axes represent monomer concentrations of each component (mM). The background surface represents the model's uncertainty, with the scalebar provided in Supplementary Figure 3B.

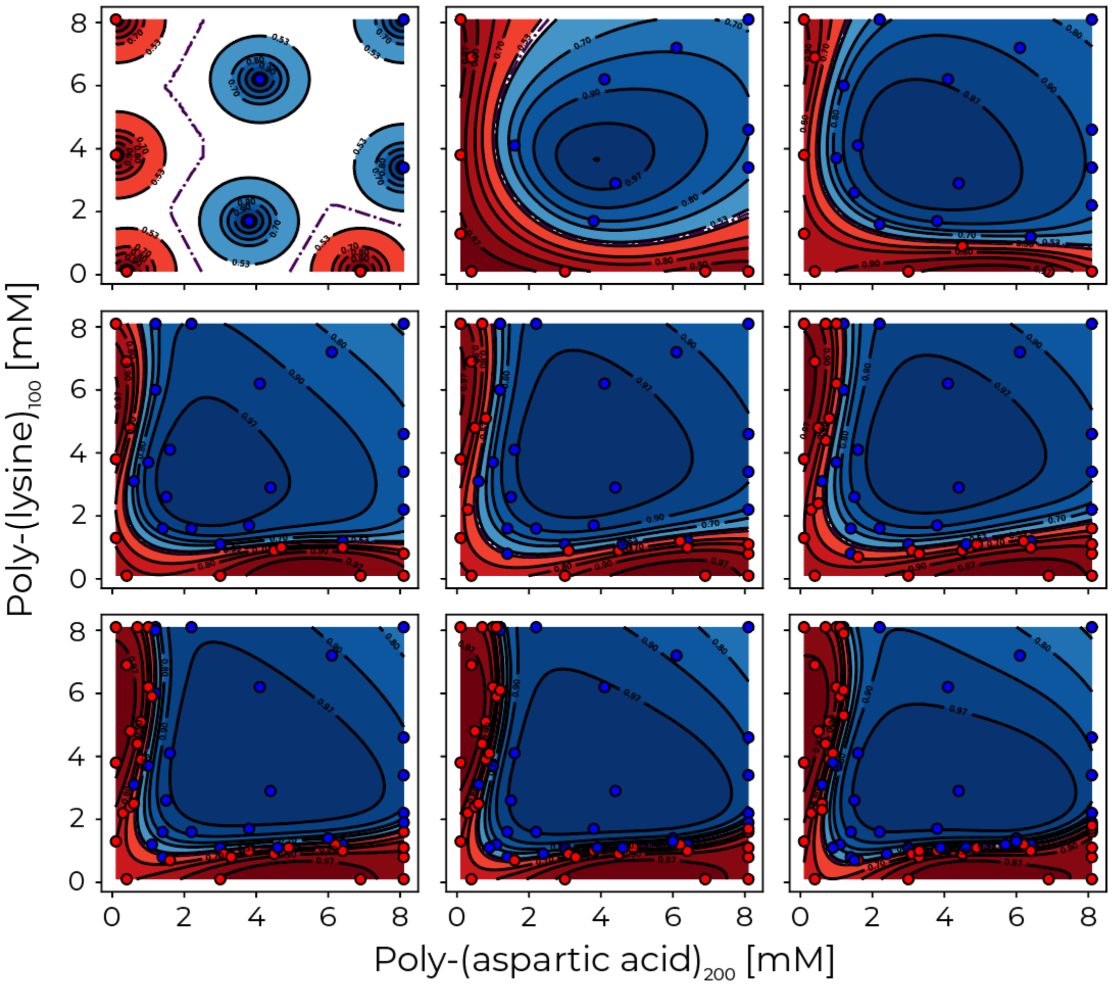

Supplementary Figure 17: Convergence of the poly-L-(lysine)$_{100}$ and poly-L-(aspartic acid)$_{200}$ condensate phase diagram (optimization experiment, Run 2). This figure shows a replicate experiment with 72 datapoints acquired over 9 cycles. The same conditions were used as in Supplementary Figure 15. Blue points represent phase separation, while red points indicate no phase separation. The corresponding entropy map is provided in Supplementary Figure 18. The background surface represents the model's predictions, with the scalebar provided in Supplementary Figure 3A. Axes represent monomer concentrations of each component (mM).

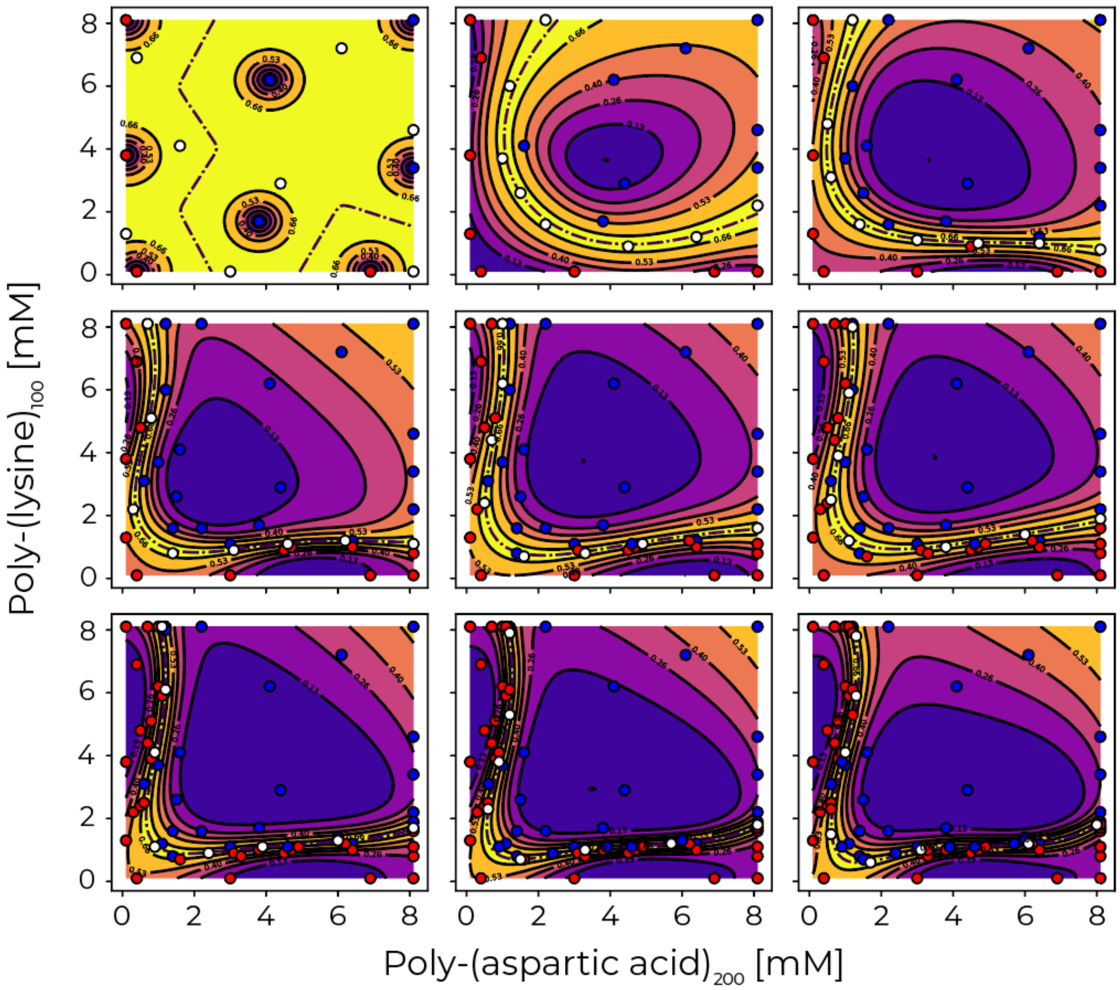

Supplementary Figure 18: Entropy map of the poly-L-(lysine)$_{100}$ and poly-L-(aspartic acid)$_{200}$ condensate phase diagram (optimization experiment, Run 2). This figure shows the entropy maps for the phase diagrams in Supplementary Figure 17. Blue points represent phase separation, red points indicate no phase separation, and white points indicate the newly requested datapoints. Axes represent monomer concentrations of each component (mM). The background surface represents the model's uncertainty, with the scalebar provided in Supplementary Figure 3B.

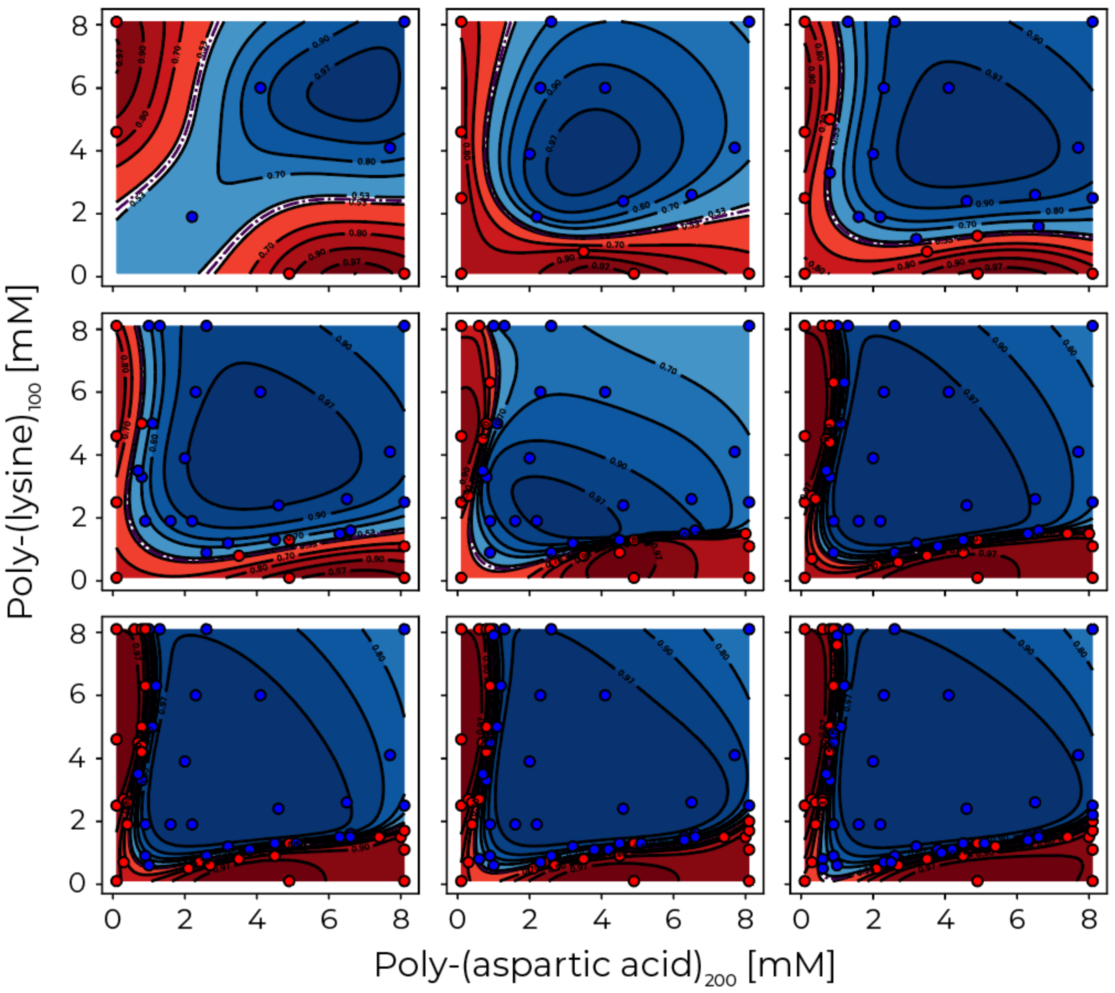

Supplementary Figure 19: Convergence of the poly-L-(lysine)$_{100}$ and poly-L-(aspartic acid)$_{200}$ condensate phase diagram (optimization experiment, Run 3). This figure shows a replicate experiment with 72 datapoints acquired over 9 cycles. The same conditions were used as in Supplementary Figure 15. Blue points represent phase separation, while red points indicate no phase separation. The corresponding entropy map is provided in Supplementary Figure 20. The background surface represents the model's predictions, with the scalebar provided in Supplementary Figure 3A. Axes represent monomer concentrations of each component (mM).

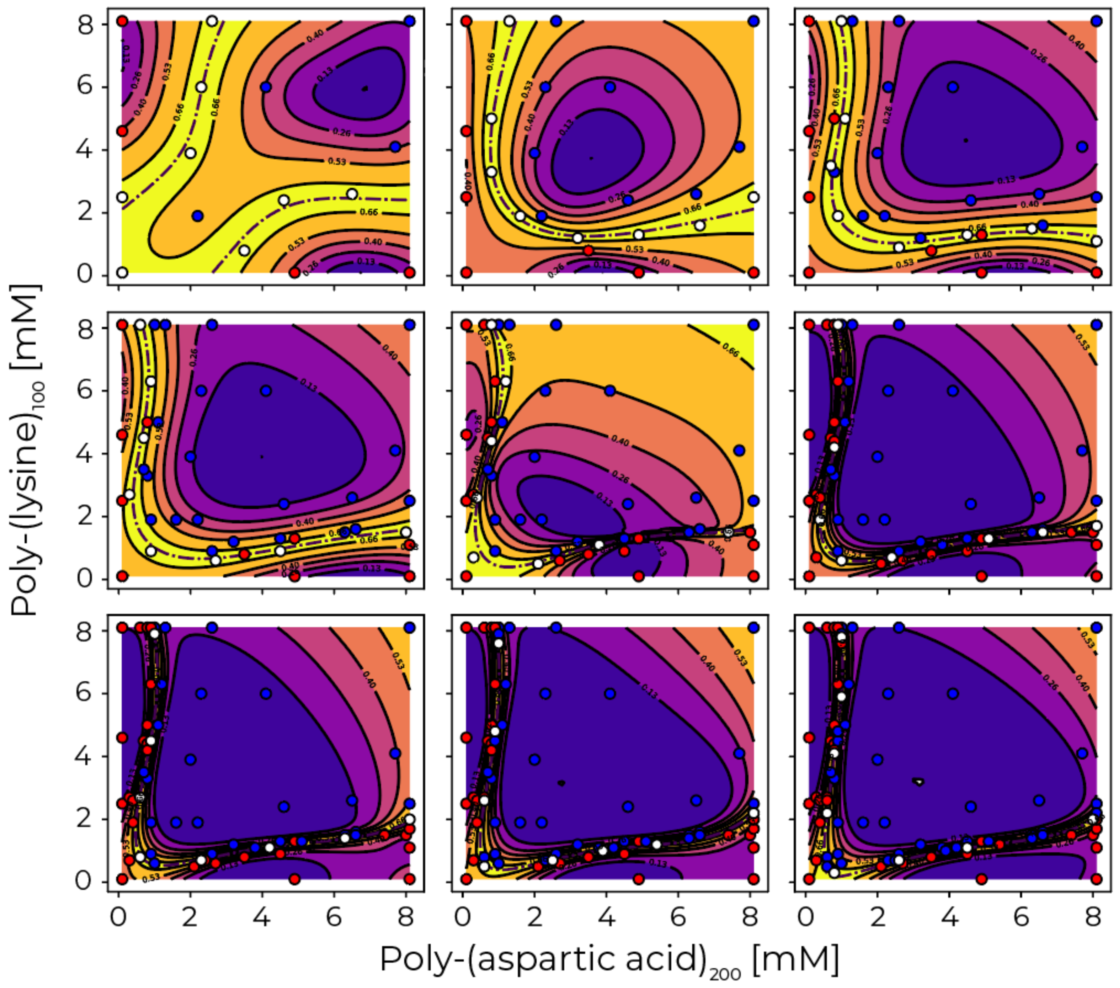

Supplementary Figure 20: Entropy map of the poly-L-(lysine)$_{100}$ and poly-L-(aspartic acid)$_{200}$ condensate phase diagram (optimization experiment, Run 3). This figure shows the entropy maps for the phase diagrams in Supplementary Figure 19. Blue points represent phase separation, red points indicate no phase separation, and white points indicate the newly requested datapoints. Axes represent monomer concentrations of each component (mM). The background surface represents the model's uncertainty, with the scalebar provided in Supplementary Figure 3B.

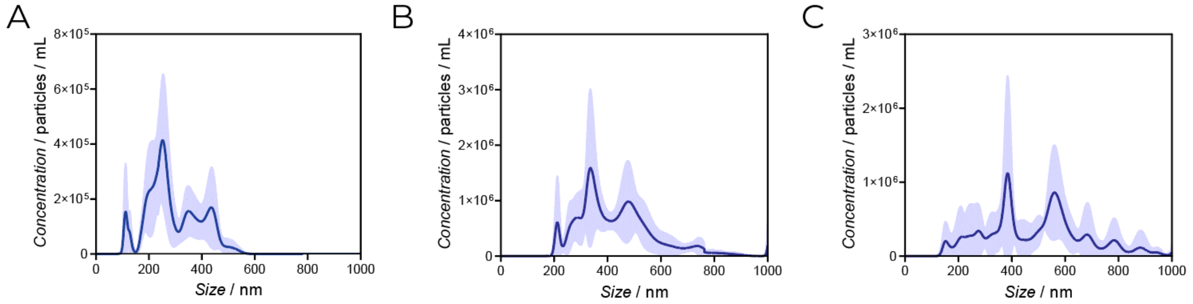

D

Conc. poly-*L*-(aspartic acid)$_{200}$ monomers [mM]	Mean particle size [nm]	Conc. particles [particles mL^{-1}; x10^{8}]	$V_{single\ coacervate}$ [ml; x10^{-14}]	Volume fraction [%]
0.1	288 +/- 11	5.6 +/- 0.7	1.25 +/- 0.14	0.0007 +/- 0.0001
0.5	436 +/- 140	35.2 +/- 3.6	4.34 +/- 4.18	0.0152 +/- 0.0147
1.0	497 +/- 167	26.3 +/- 2.7	6.43 +/- 6.48	0.0169 +/- 0.0171

Supplementary Figure 21: Nanoparticle Tracking Analysis (NTA) of coacervates formed from poly-*L*-(lysine)$_{100}$ / poly-*L*-(aspartic acid)$_{200}$. (**A–C**) Particle size distributions showing concentration per size bin for samples containing: (**A**) 0.1 mM, (**B**) 0.5 mM, and (**C**) 1.0 mM poly-*L*-(aspartic acid)$_{200}$. In all cases, poly-*L*-(lysine)$_{100}$ was fixed at 2.0 mM. (D) Summary of key coacervate properties as determined by NTA, including mean particle size, particle concentration, estimated single-particle volume, and total coacervate volume fraction per condition. Polypeptide concentrations are reported in monomer concentrations.

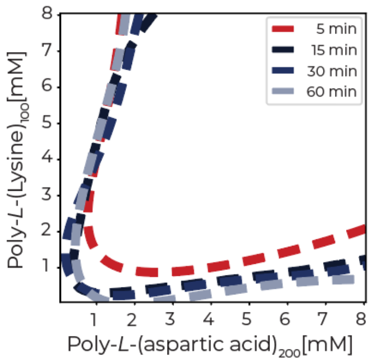

Supplementary Figure 22: Time-resolved phase diagram mapping of poly-*L*-(lysine)$_{100}$ / poly-*L*-(aspartic acid)$_{200}$ coacervates. Phase diagrams were constructed by imaging after incubation times of 5, 15, 30, and 60 minutes. Each condition was treated as a separate experiment. The operational phase boundaries are shown as dashed lines for each time point. Substantial shifts in the phase boundary are observed between 5 and 15 minutes, indicating ongoing droplet settling. Beyond 15 minutes, the phase boundary remains stable, suggesting that the system has reached a near-equilibrium state under the experimental conditions. Polypeptide concentrations are reported in monomer concentrations.

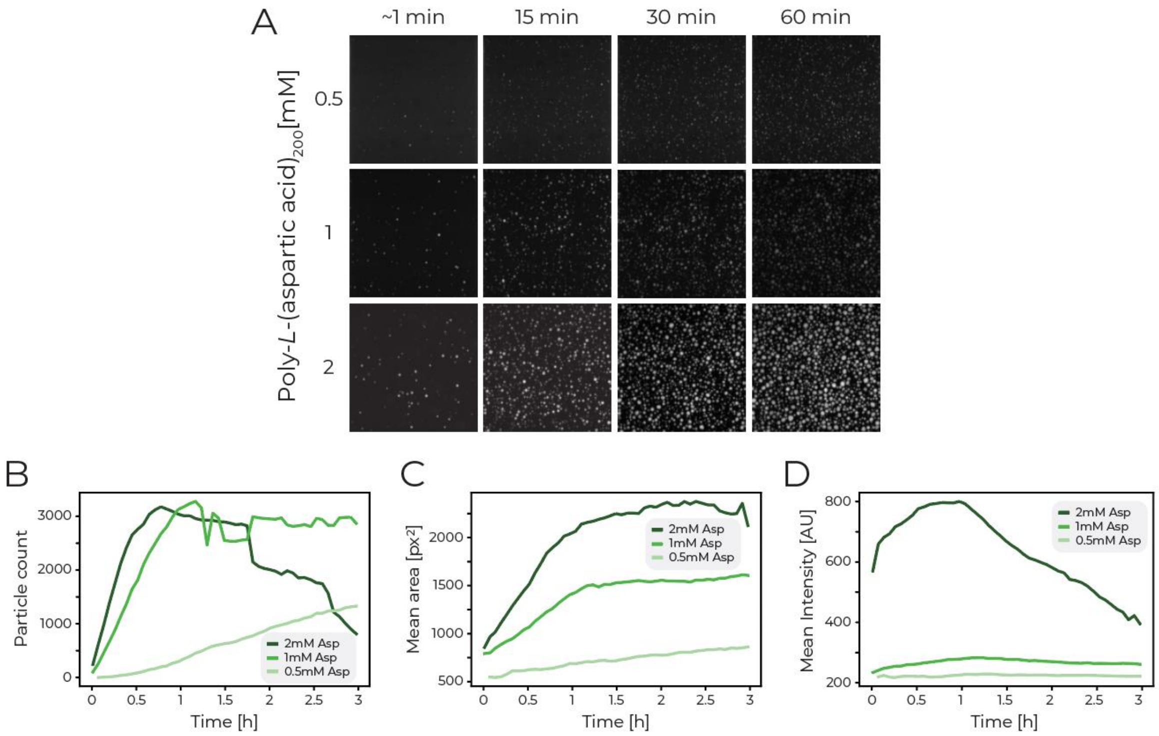

Supplementary Figure 23: Time-resolved mapping of coacervate properties in poly-L-(lysine)$_{100}$ and poly-L-(aspartic acid)$_{200}$ formulations. (**A**) Representative confocal micrographs of three selected formulations containing 2.0 mM poly-L-(lysine)$_{100}$ combined with 0.5, 1.0, or 2.0 mM poly-L-(aspartic acid)$_{200}$. The shown micrographs were taking at approximately 1, 15, 30, and 60 minutes after mixing. (**B-D**) Quantitative analysis of condensate properties for the same formulations over time: total particle count (**B**), mean particle area (**C**), and mean fluorescence intensity (**D**). These measurements reveal characteristic LLPS dynamics, including initial coalescence, settling into the imaging plane, and time-dependent photobleaching of fluorescent signal due to repeated imaging. Polypeptide concentrations are reported in monomer concentrations.

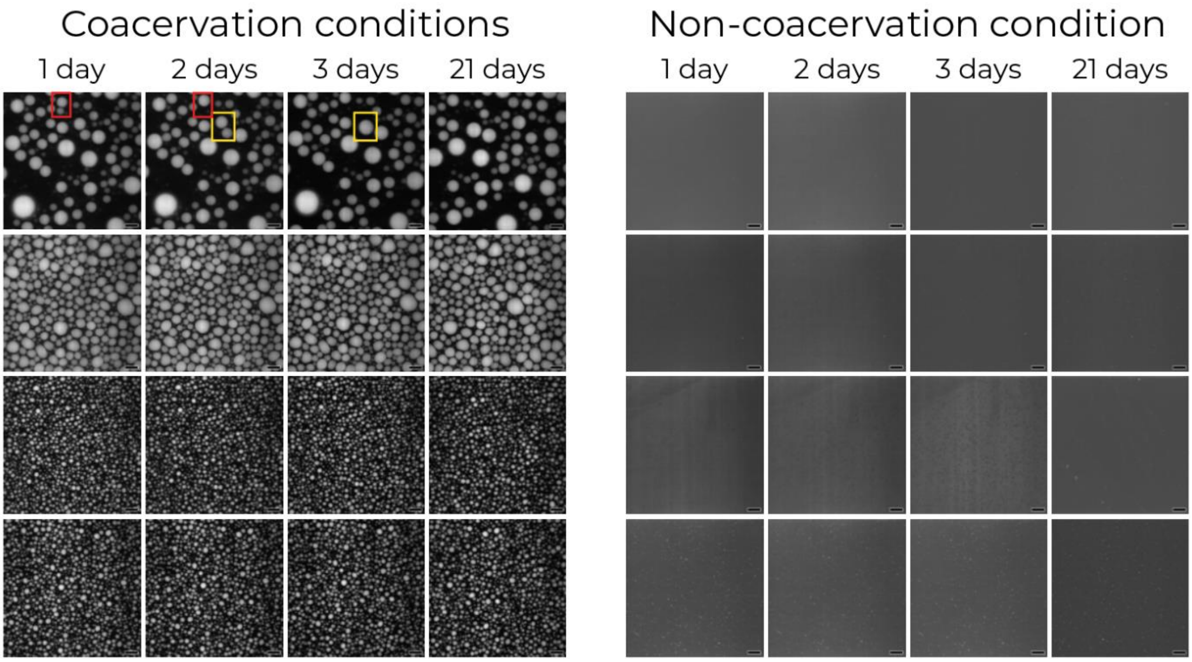

Supplementary Figure 24: Time-lapse imaging of coacervates formed by poly-*L*-(lysine)$_{100}$ and poly-*L*-(aspartic acid)$_{200}$. Confocal micrographs show selected formulations with distinct coacervate morphologies alongside a non-phase-separating control panel, imaged at 1 day, 2 days, 3 days, and 21 days after sample preparation. Samples were stored at room temperature in a sealed microplate and re-imaged over time by repeatedly moving the plate in and out of the microscope. A slight drift in field of view is visible at later time points due to repositioning. Across all coacervation conditions, droplets remained clearly visible and retained their morphology over three weeks, with no signs of structural maturation or solidification. Colored boxes highlight selected droplet fusion events over time. For transparency, micrographs were contrast-enhanced to allow visualization of faint background signals in the non-coacervating samples and to confirm the absence of phase separation. Scale bars: 20 µm.

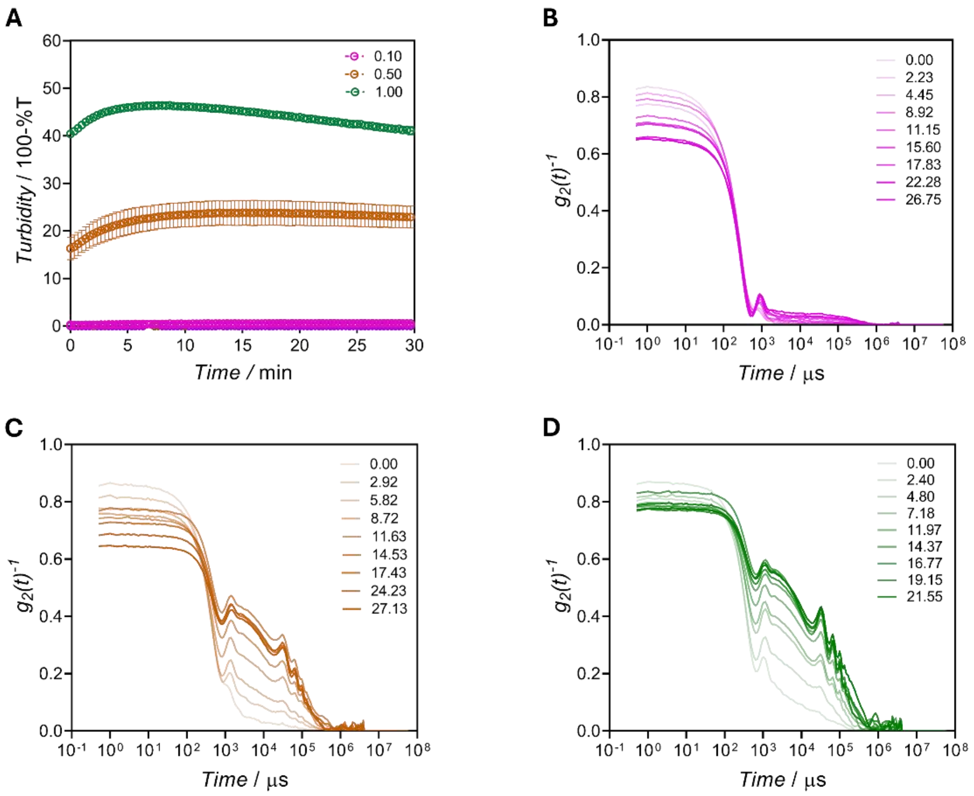

Supplementary Figure 25: Time-resolved measurements validating the 15-minute incubation time used in the automated pipeline. (**A**) Turbidity kinetics of poly-*L*-(lysine)$_{100}$ / poly-*L*-(aspartic acid)$_{200}$ formulations near the phase boundary. Poly-*L*-(lysine)$_{100}$ was fixed at 2.00 mM, while poly-*L*-(aspartic acid)$_{200}$ was varied from 0.1 to 1.00 mM. (**B**-**D**) Time correlation functions obtained by dynamic light scattering (DLS) for the same formulations, measured between 0 and 25 minutes post-mixing (timepoints indicated in the legend). The decay of the autocorrelation function indicates the diffusion behavior of the coacervates, which is linked to particle size via the Stokes-Einstein equation. These correlograms, particularly in panels C and D, reveal a progressive flattening that reflects changes in particle size distribution and droplet growth over time. This flattening plateaued around 15 minutes for each sample, supporting the use of the 15 minute-mark as a consistent and practical snapshot for coacervate quantification. Polypeptide concentrations are reported in monomer concentrations.

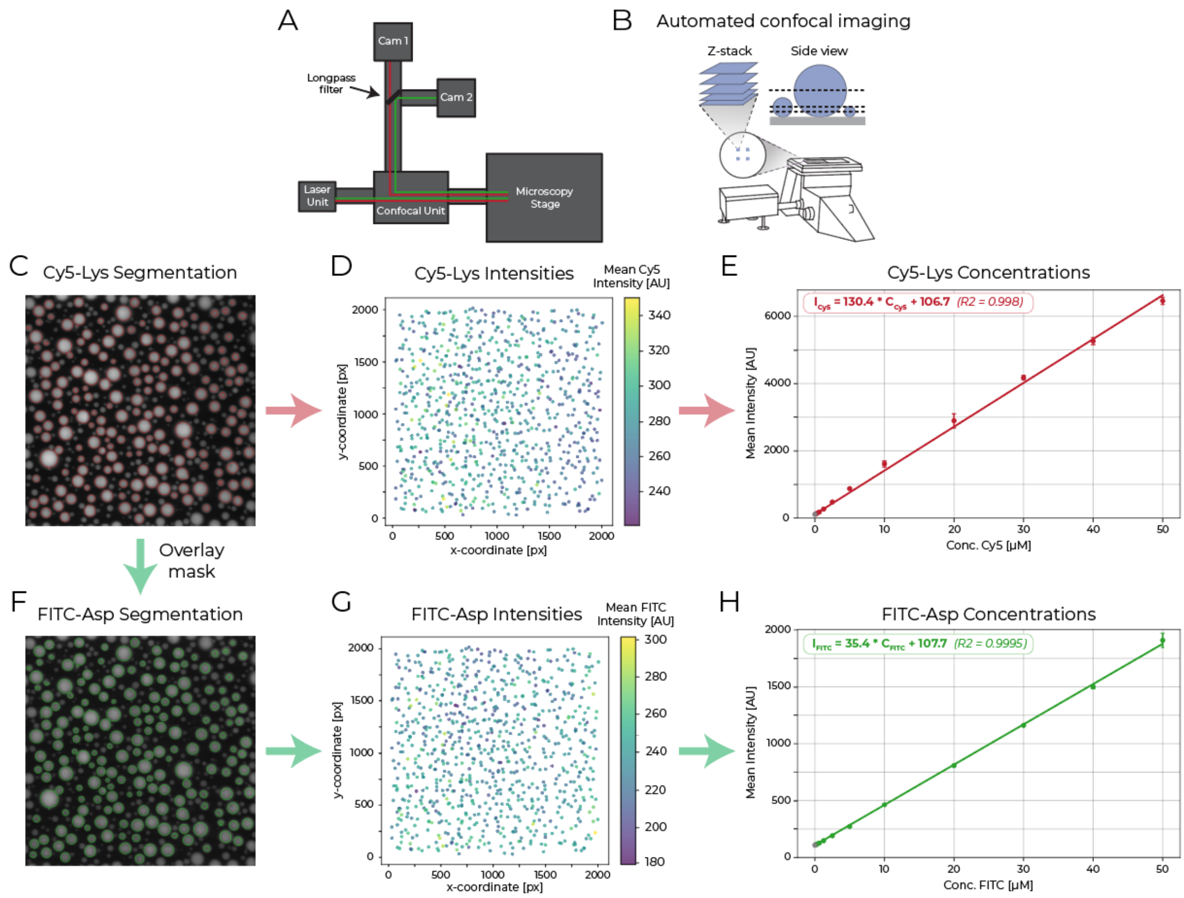

Supplementary Figure 26: Measurement of dense-phase dye concentrations using dual-color confocal imaging. (**A**) Schematic of the confocal imaging setup with dual-camera acquisition. A longpass filter splits emission signals, enabling simultaneous imaging of FITC (488 nm) and Cy5 (640 nm) channels. (**B**) Automated z-stack acquisition from coacervate samples, capturing condensates across multiple focal planes. (**C**) Example segmentation of the Cy5 channel, with detected condensates outlined in red. (**D**) Spatial map of all detected Cy5-positive particles across four grid positions and z-planes, color-coded by mean Cy5 fluorescence intensity. (**E**) Calibration curve relating Cy5 fluorescence intensity to known Cy5 concentrations (R^2 = 0.998), enabling conversion of mean intensity values to local dye concentrations. (**F**) Cy5 mask from panel (**C**) overlaid on the FITC channel to extract FITC signal from the same particles, shown with green outlines. (**G**) Spatial map showing the corresponding FITC intensity values for each segmented region. (**H**) Calibration curve for FITC fluorescence versus concentration (R^2 = 0.9995), enabling quantification of local FITC concentration.

Data shown correspond to a sample containing 6.1 mM poly-*L*-(lysine)$_{100}$ and 8.1 mM poly-*L*-(aspartic acid)$_{200}$ (monomer concentrations), each spiked with 250 nM of fluorescently labeled polypeptides. The resulting dense-phase concentrations were estimated to be $[Cy5]_{dense}$ = 1.3 ± 0.2 µM and $[FITC]_{dense}$ = 3.6 ± 0.5 µM.

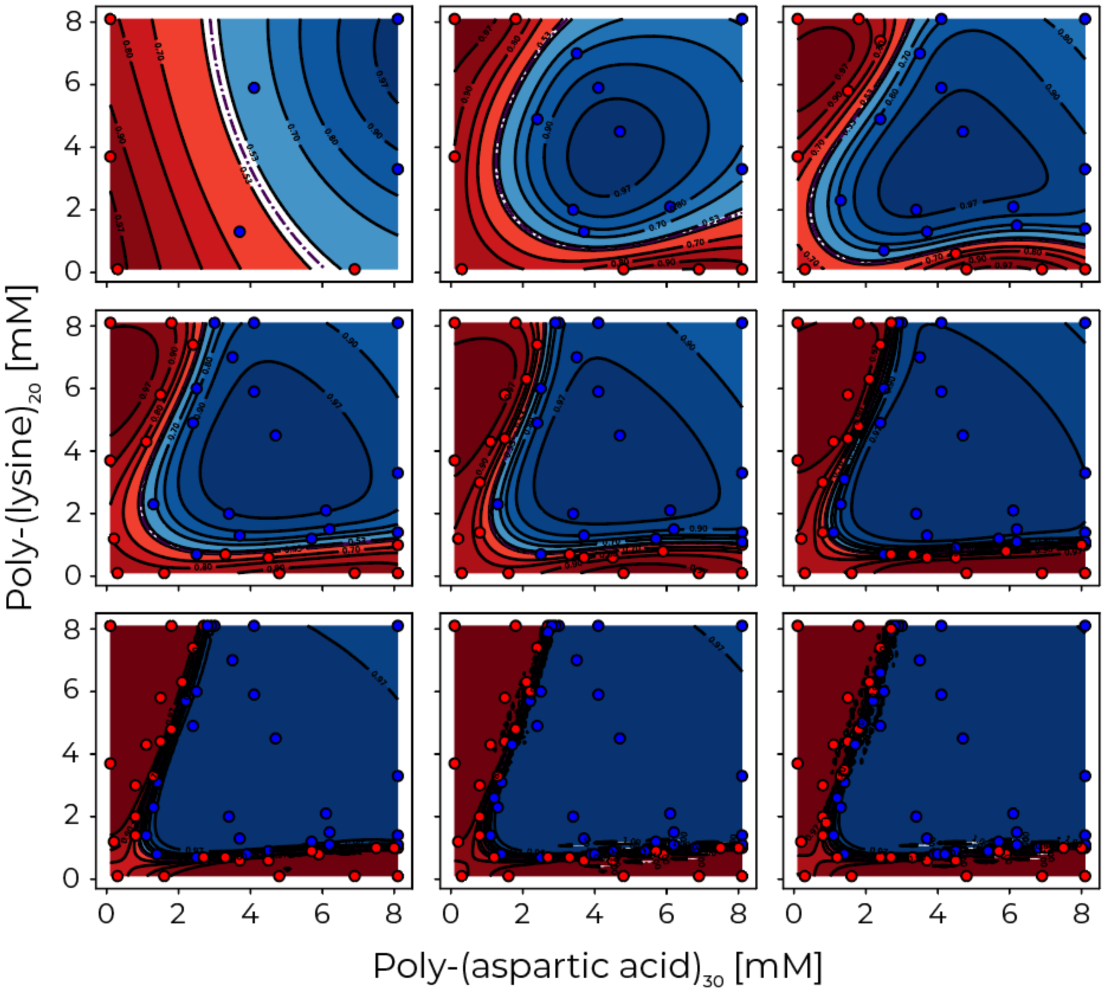

Supplementary Figure 27: Convergence of the poly-L-(lysine)$_{20}$ and poly-L-(aspartic acid)$_{30}$ condensate phase diagram. This figure shows a 72 datapoints acquired over 9 cycles. The same conditions were used as in Supplementary Figure 4 and the final phase diagram is depicted in main text Figure 5. Blue points represent phase separation, while red points indicate no phase separation. The corresponding entropy map is provided in Supplementary Figure 28. The background surface represents the model's predictions, with the scalebar provided in Supplementary Figure 3A. Axes represent monomer concentrations of each component (mM).

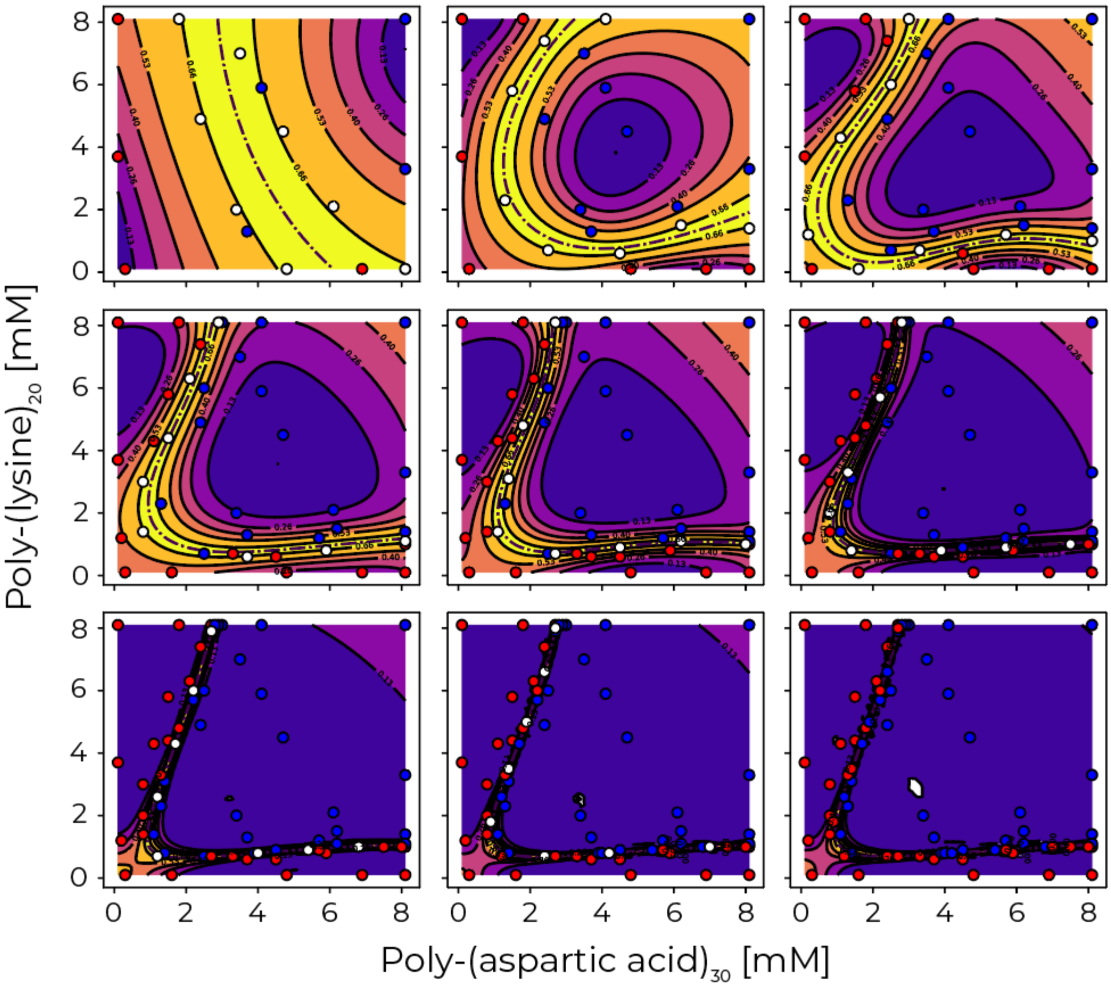

Supplementary Figure 28: Entropy map of the poly-L-(lysine)$_{20}$ and poly-L-(aspartic acid)$_{30}$ condensate phase diagram. This figure shows the entropy maps for the phase diagrams in Supplementary Figure 27. Blue points represent phase separation, red points indicate no phase separation, and white points indicate the newly requested datapoints. Axes represent monomer concentrations of each component (mM). The background surface represents the model's uncertainty, with the scalebar provided in Supplementary Figure 3B.

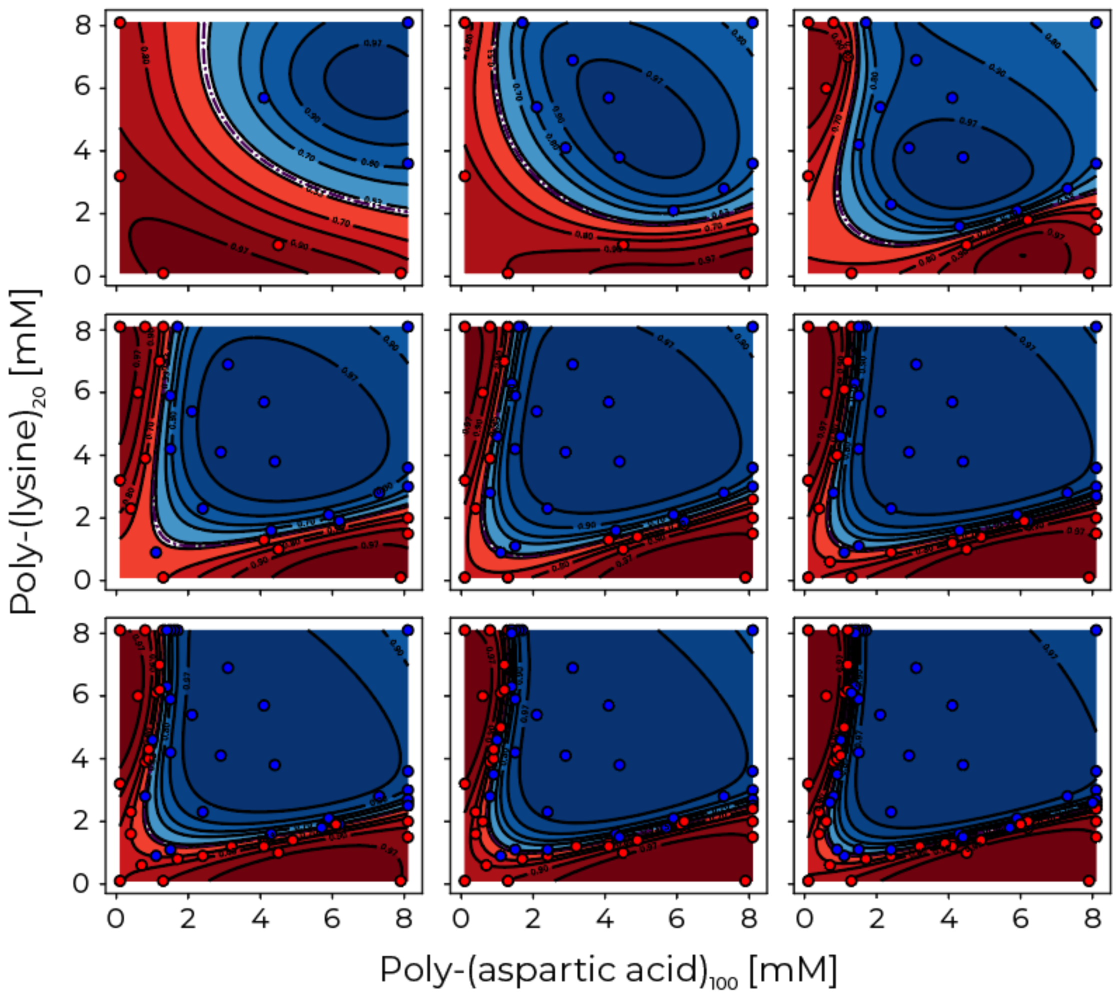

Supplementary Figure 29: Convergence of the poly-L-(lysine)$_{20}$ and poly-L-(aspartic acid)$_{100}$ condensate phase diagram. This figure shows a 72 datapoints acquired over 9 cycles. The same conditions were used as in Supplementary Figure 4 and the final phase diagram is depicted in main text Figure 5. Blue points represent phase separation, while red points indicate no phase separation. The corresponding entropy map is provided in Supplementary Figure 30. The background surface represents the model's predictions, with the scalebar provided in Supplementary Figure 3A. Axes represent monomer concentrations of each component (mM).

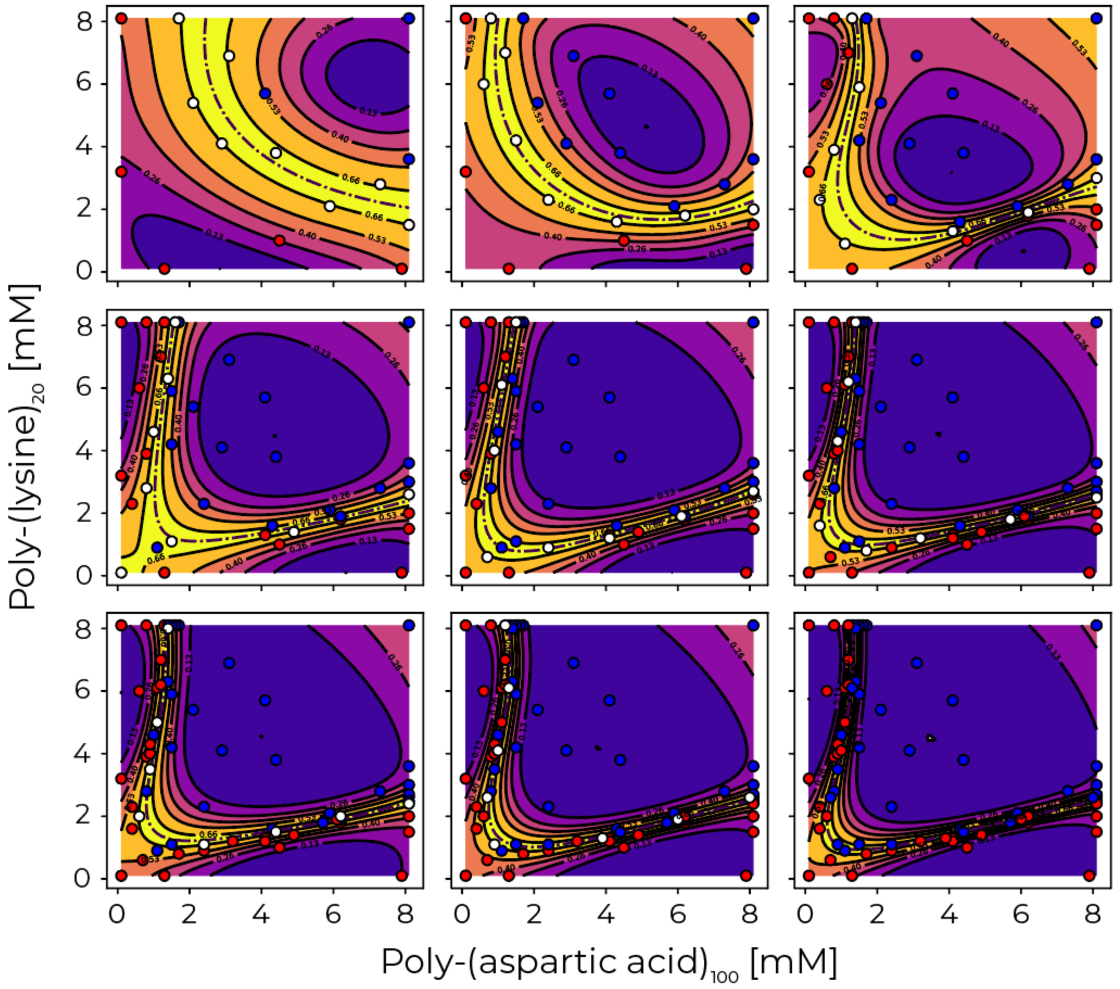

Supplementary Figure 30: Entropy map of the poly-L-(lysine)$_{20}$ and poly-L-(aspartic acid)$_{100}$ condensate phase diagram. This figure shows the entropy maps for the phase diagrams in Supplementary Figure 29. Blue points represent phase separation, red points indicate no phase separation, and white points indicate the newly requested datapoints. Axes represent monomer concentrations of each component (mM). The background surface represents the model's uncertainty, with the scalebar provided in Supplementary Figure 3B.

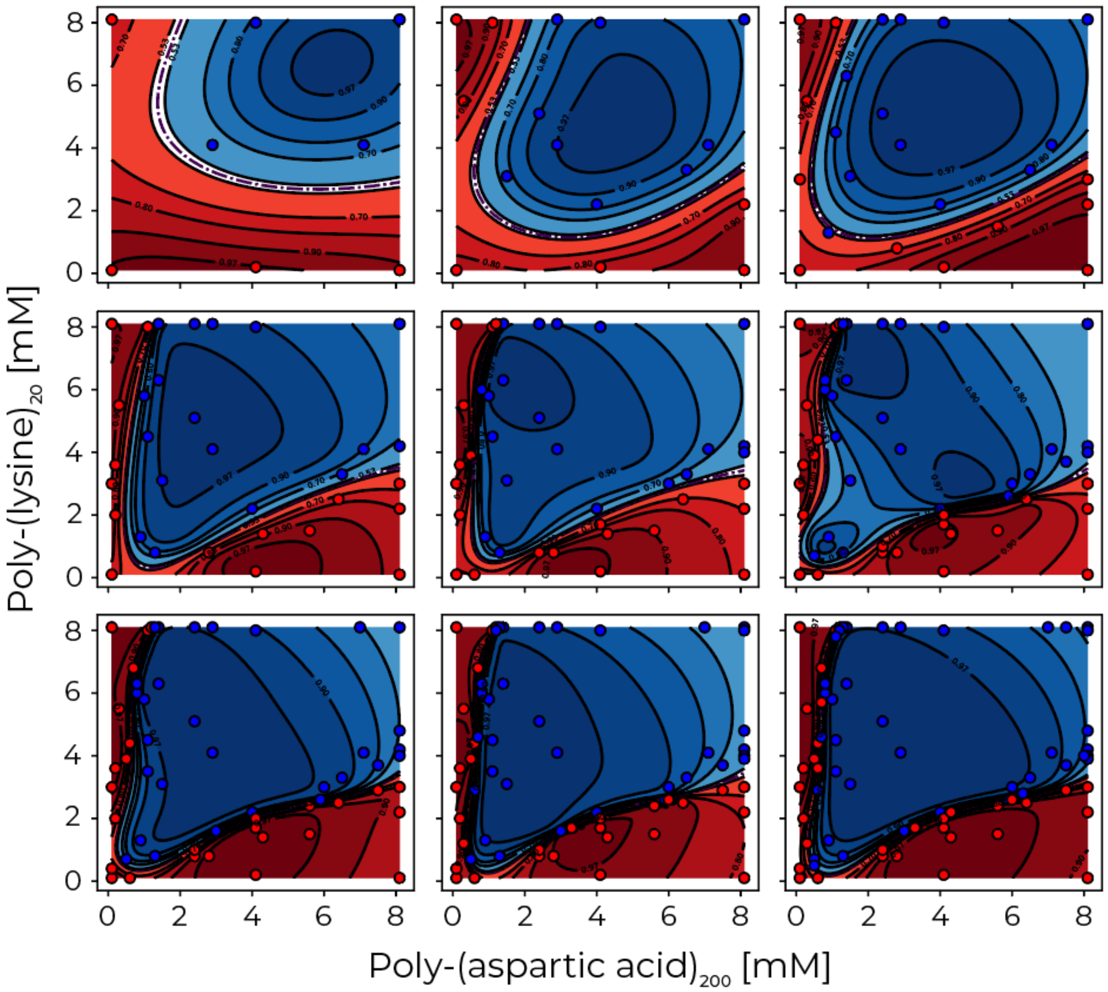

Supplementary Figure 31: Convergence of the poly-L-(lysine)$_{20}$ and poly-L-(aspartic acid)$_{200}$ condensate phase diagram. This figure shows a 72 datapoints acquired over 9 cycles. The same conditions were used as in Supplementary Figure 4 and the final phase diagram is depicted in main text Figure 5. Blue points represent phase separation, while red points indicate no phase separation. The corresponding entropy map is provided in Supplementary Figure 32. The background surface represents the model's predictions, with the scalebar provided in Supplementary Figure 3A. Axes represent monomer concentrations of each component (mM).

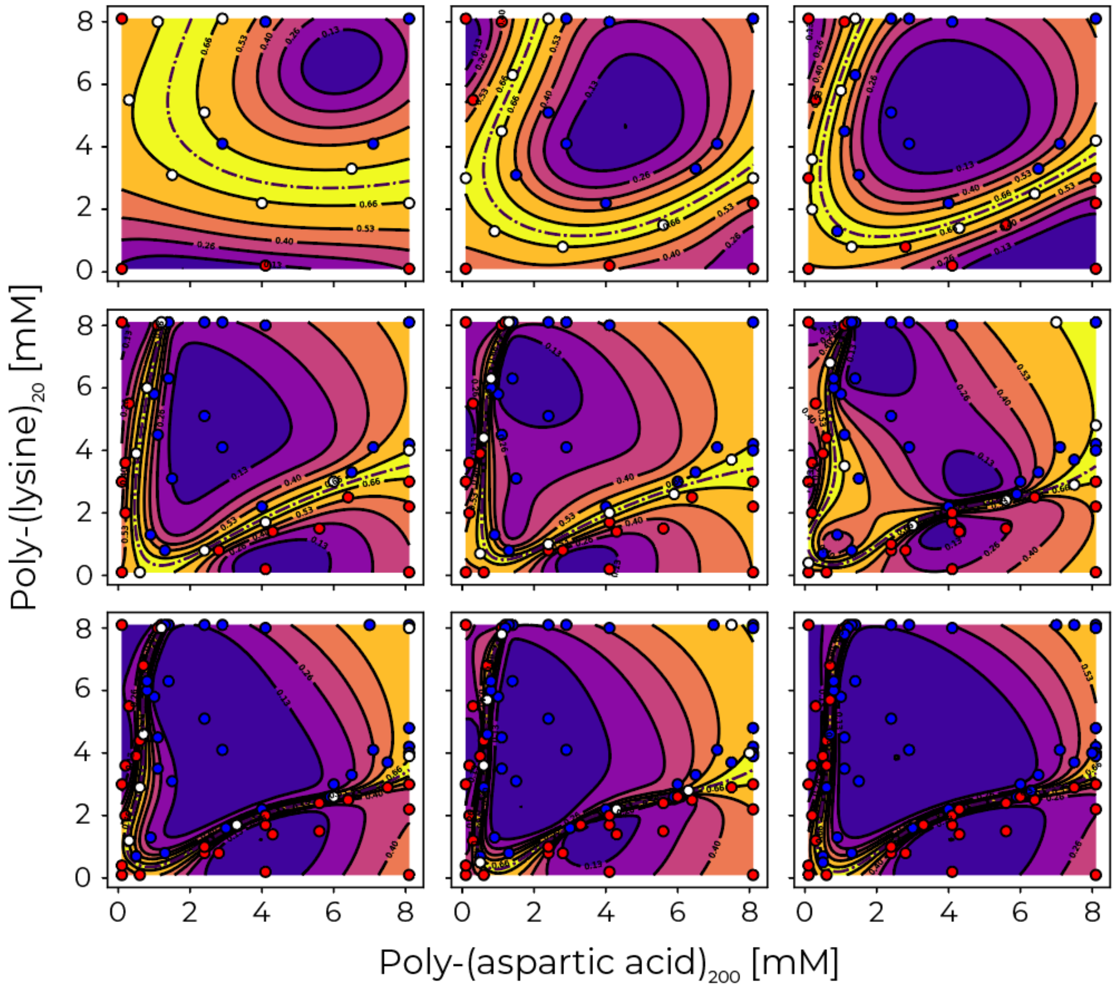

Supplementary Figure 32: Entropy map of the poly-L-(lysine)$_{20}$ and poly-L-(aspartic acid)$_{200}$ condensate phase diagram. This figure shows the entropy maps for the phase diagrams in Supplementary Figure 31. Blue points represent phase separation, red points indicate no phase separation, and white points indicate the newly requested datapoints. Axes represent monomer concentrations of each component (mM). The background surface represents the model's uncertainty, with the scalebar provided in Supplementary Figure 3B.

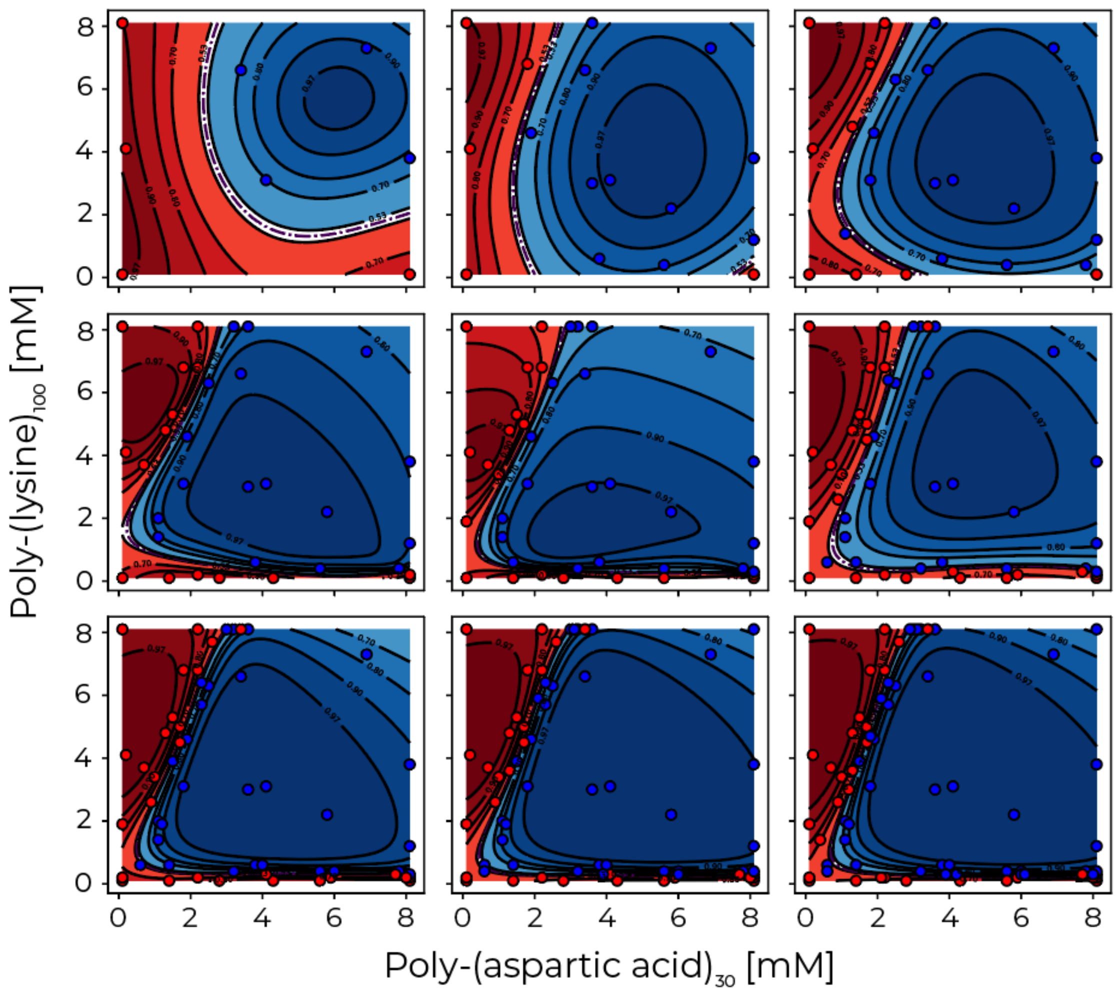

Supplementary Figure 33: Convergence of the poly-L-(lysine)$_{100}$ and poly-L-(aspartic acid)$_{30}$ condensate phase diagram. This figure shows a 72 datapoints acquired over 9 cycles. The same conditions were used as in Supplementary Figure 4 and the final phase diagram is depicted in main text Figure 5. Blue points represent phase separation, while red points indicate no phase separation. The corresponding entropy map is provided in Supplementary Figure 34. The background surface represents the model's predictions, with the scalebar provided in Supplementary Figure 3A. Axes represent monomer concentrations of each component (mM).

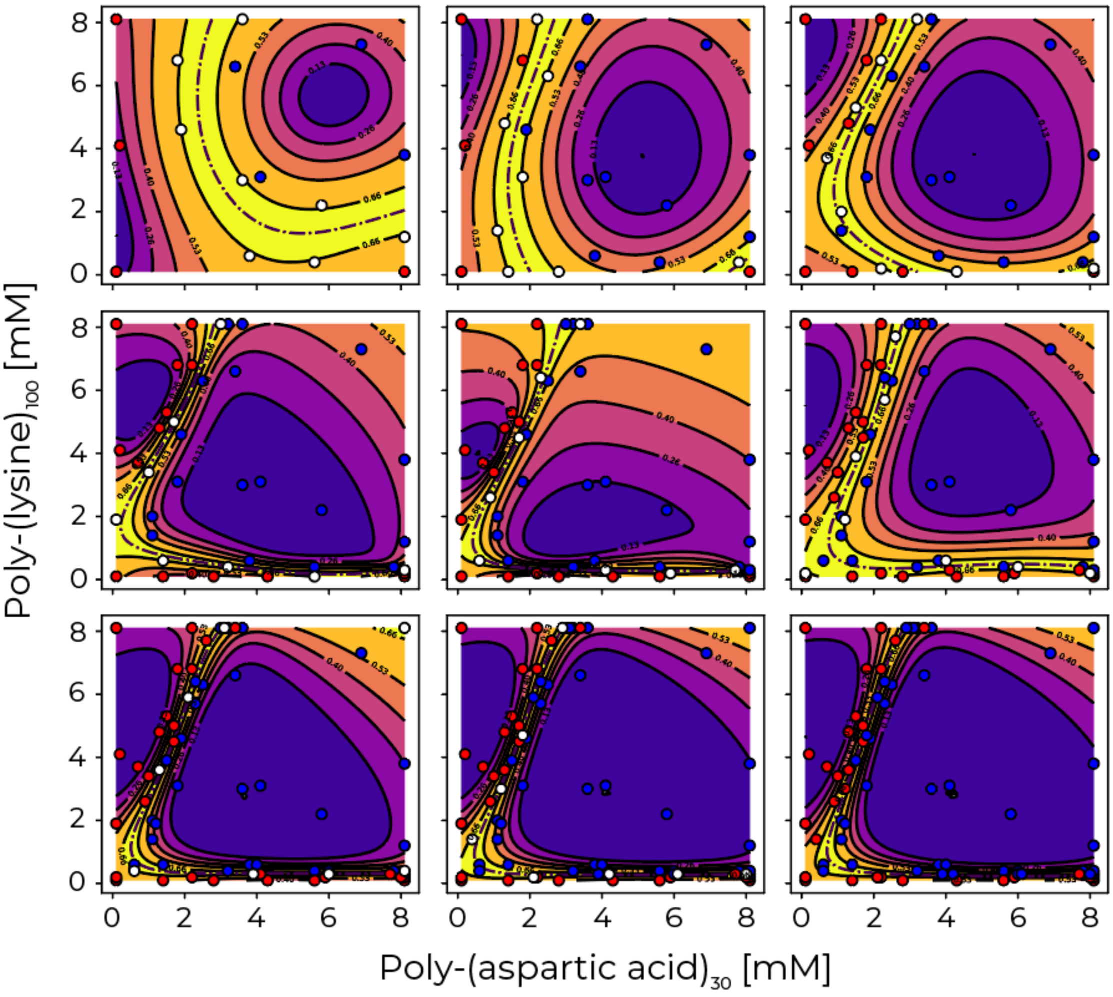

Supplementary Figure 34: Entropy map of the poly-L-(lysine)$_{100}$ and poly-L-(aspartic acid)$_{30}$ condensate phase diagram. This figure shows the entropy maps for the phase diagrams in Supplementary Figure 33. Blue points represent phase separation, red points indicate no phase separation, and white points indicate the newly requested datapoints. Axes represent monomer concentrations of each component (mM). The background surface represents the model's uncertainty, with the scalebar provided in Supplementary Figure 3B.

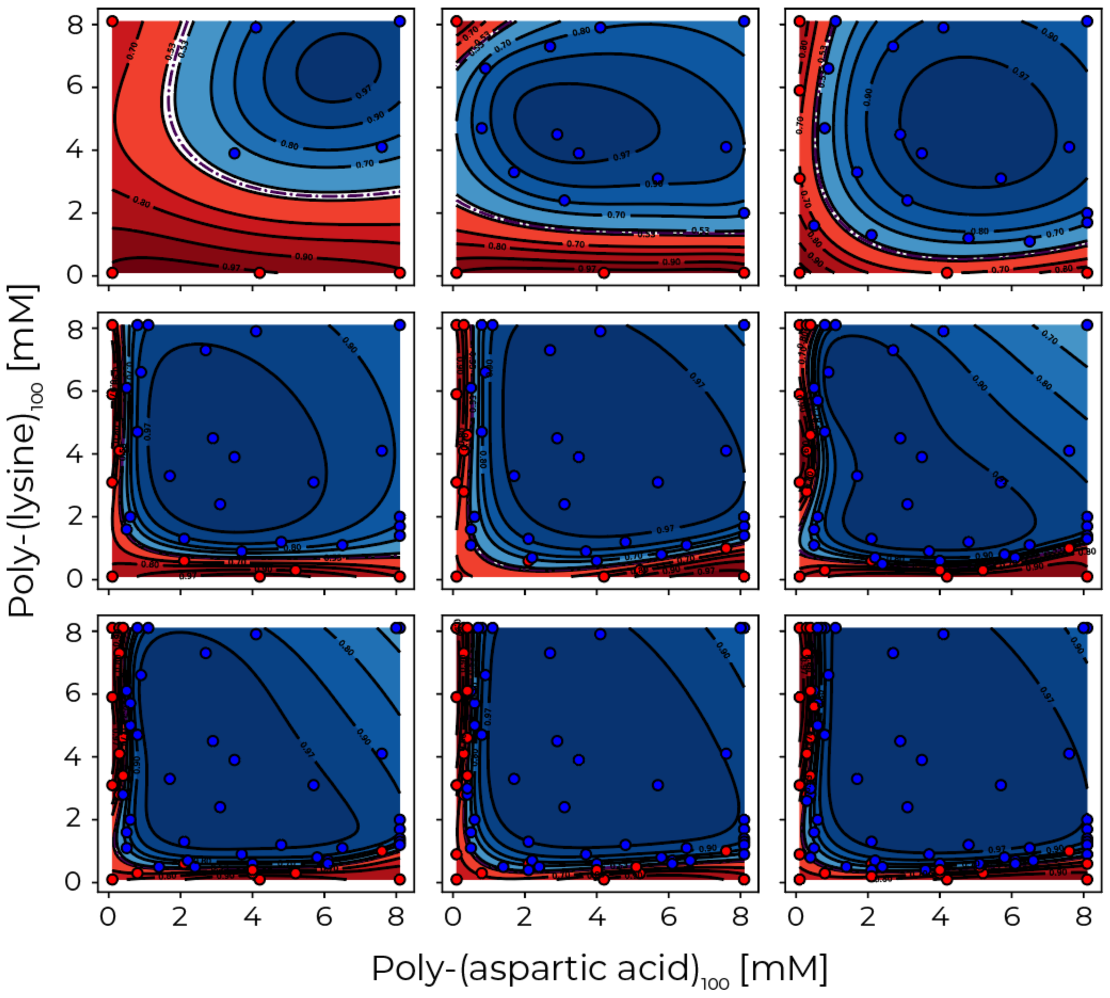

Supplementary Figure 35: Convergence of the poly-L-(lysine)$_{100}$ and poly-L-(aspartic acid)$_{100}$ condensate phase diagram. This figure shows a 72 datapoints acquired over 9 cycles. The same conditions were used as in Supplementary Figure 4 and the final phase diagram is depicted in main text Figure 5. Blue points represent phase separation, while red points indicate no phase separation. The corresponding entropy map is provided in Supplementary Figure 36. The background surface represents the model's predictions, with the scalebar provided in Supplementary Figure 3A. Axes represent monomer concentrations of each component (mM).

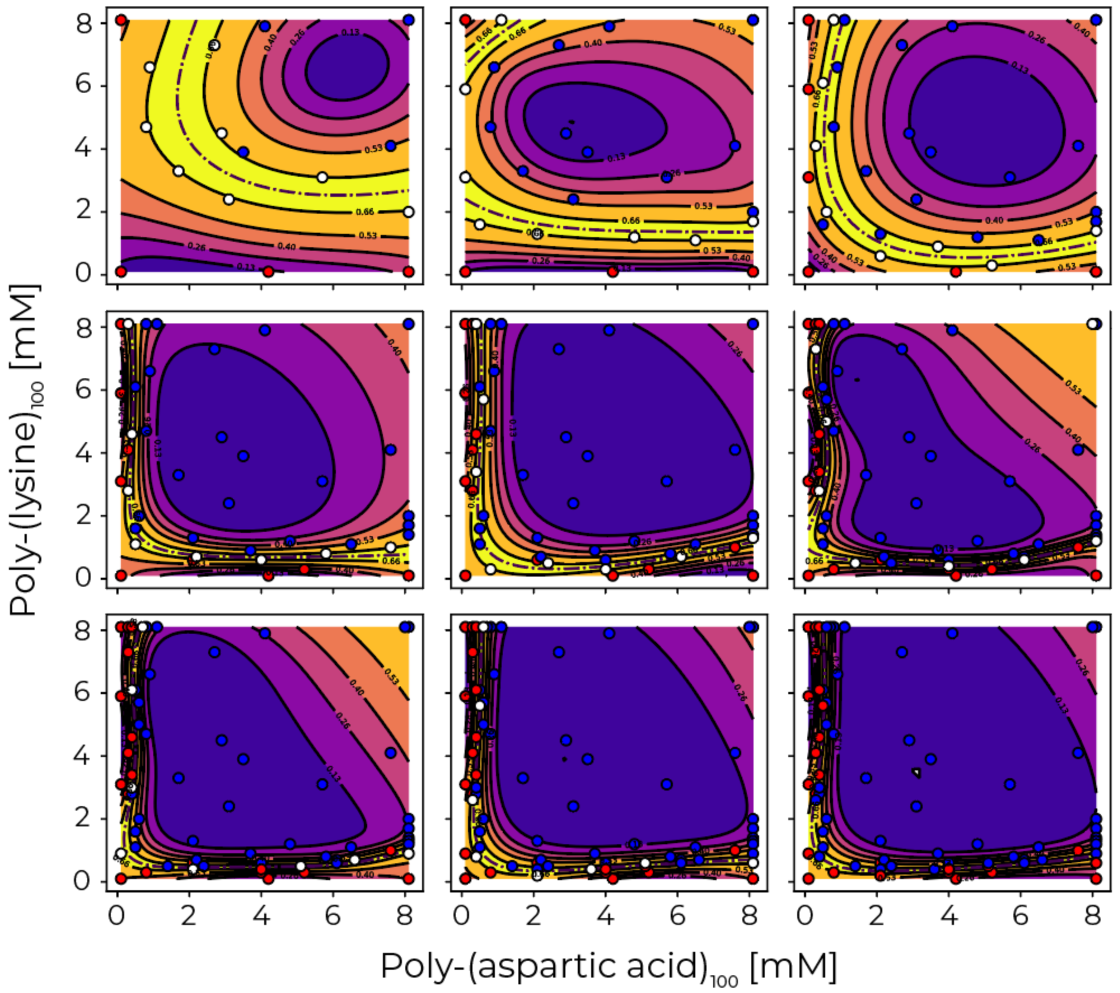

Supplementary Figure 36: Entropy map of the poly-L-(lysine)$_{100}$ and poly-L-(aspartic acid)$_{100}$ condensate phase diagram. This figure shows the entropy maps for the phase diagrams in Supplementary Figure 35. Blue points represent phase separation, red points indicate no phase separation, and white points indicate the newly requested datapoints. Axes represent monomer concentrations of each component (mM). The background surface represents the model's uncertainty, with the scalebar provided in Supplementary Figure 3B.

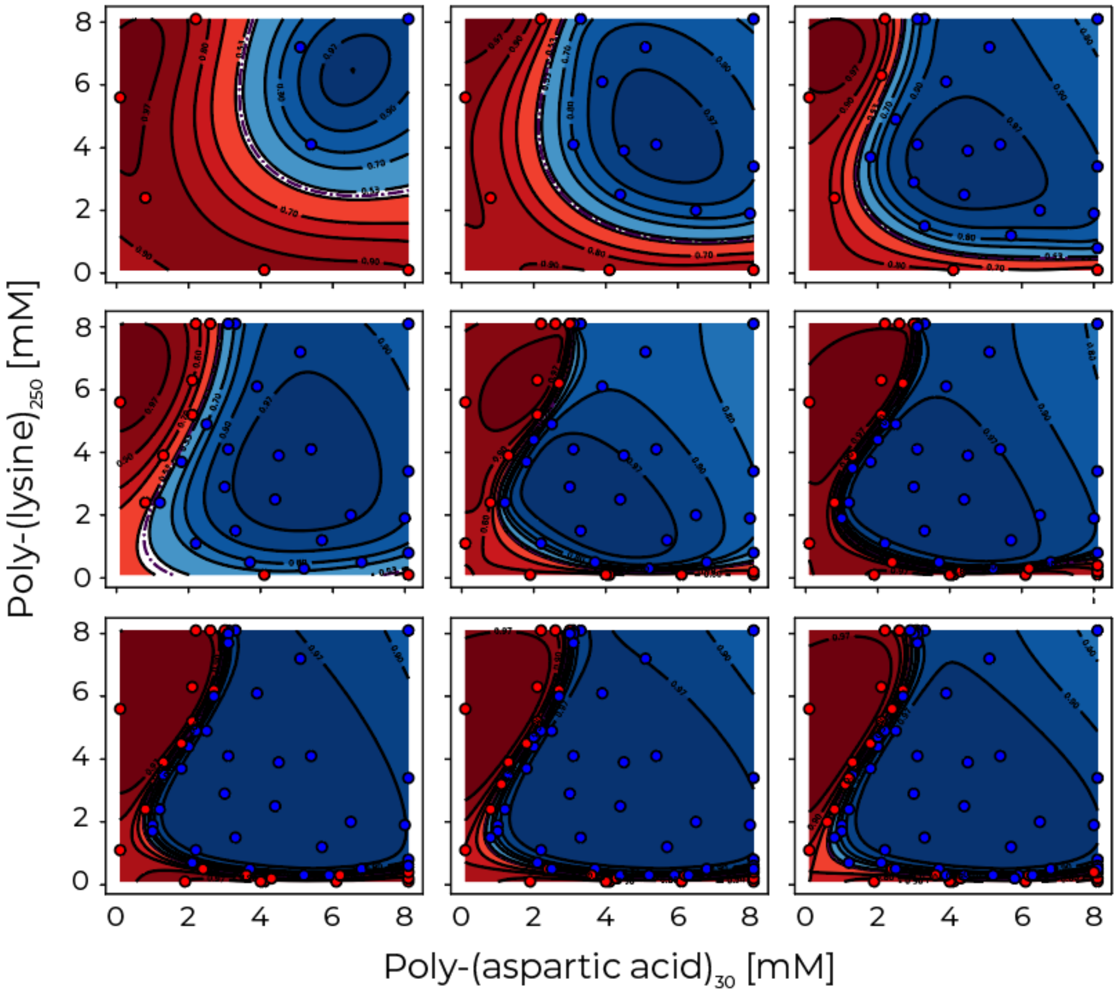

Supplementary Figure 37: Convergence of the poly-L-(lysine)$_{250}$ and poly-L-(aspartic acid)$_{30}$ condensate phase diagram. This figure shows a 72 datapoints acquired over 9 cycles. The same conditions were used as in Supplementary Figure 4 and the final phase diagram is depicted in main text Figure 5. Blue points represent phase separation, while red points indicate no phase separation. The corresponding entropy map is provided in Supplementary Figure 38. The background surface represents the model's predictions, with the scalebar provided in Supplementary Figure 3A. Axes represent monomer concentrations of each component (mM).

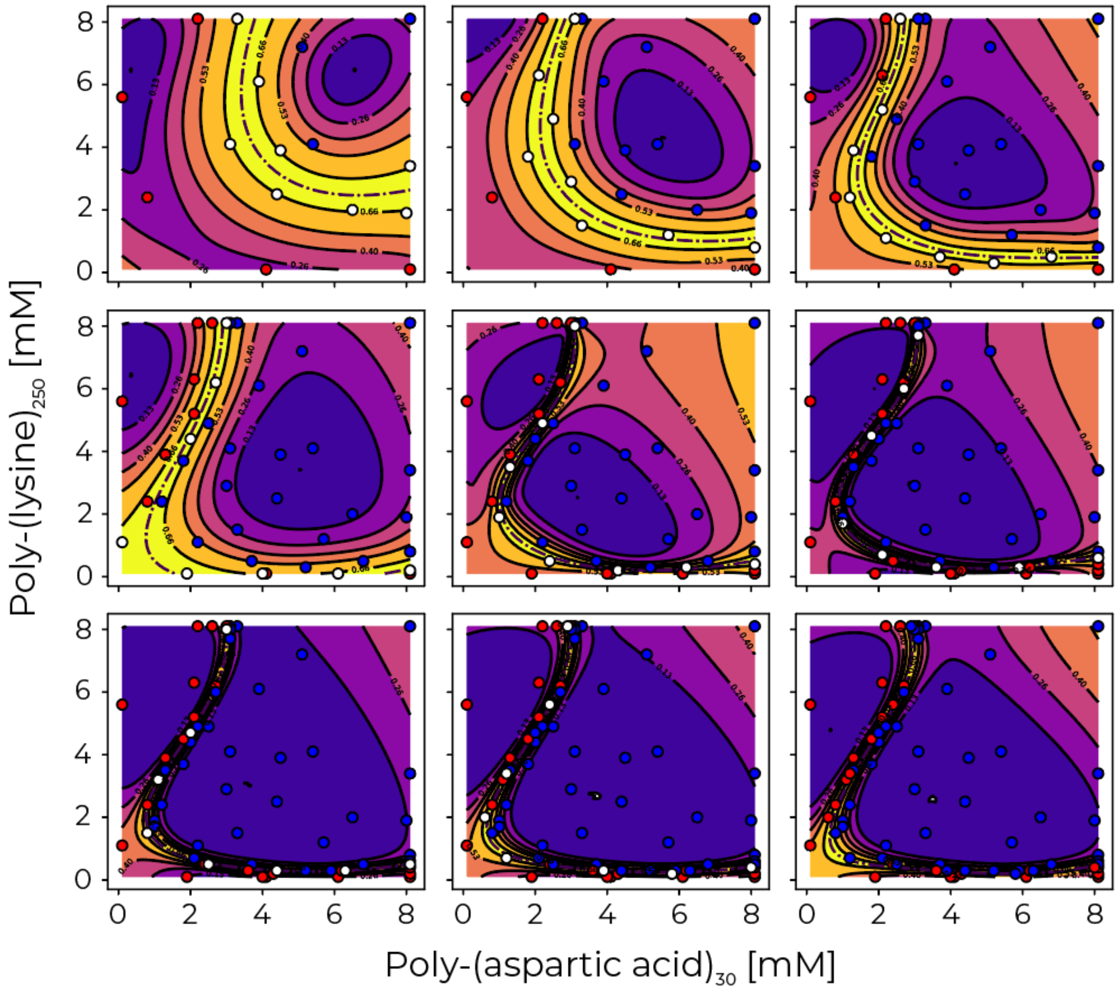

Supplementary Figure 38: Entropy map of the poly-L-(lysine)$_{250}$ and poly-L-(aspartic acid)$_{30}$ condensate phase diagram. This figure shows the entropy maps for the phase diagrams in Supplementary Figure 37. Blue points represent phase separation, red points indicate no phase separation, and white points indicate the newly requested datapoints. Axes represent monomer concentrations of each component (mM). The background surface represents the model's uncertainty, with the scalebar provided in Supplementary Figure 3B.

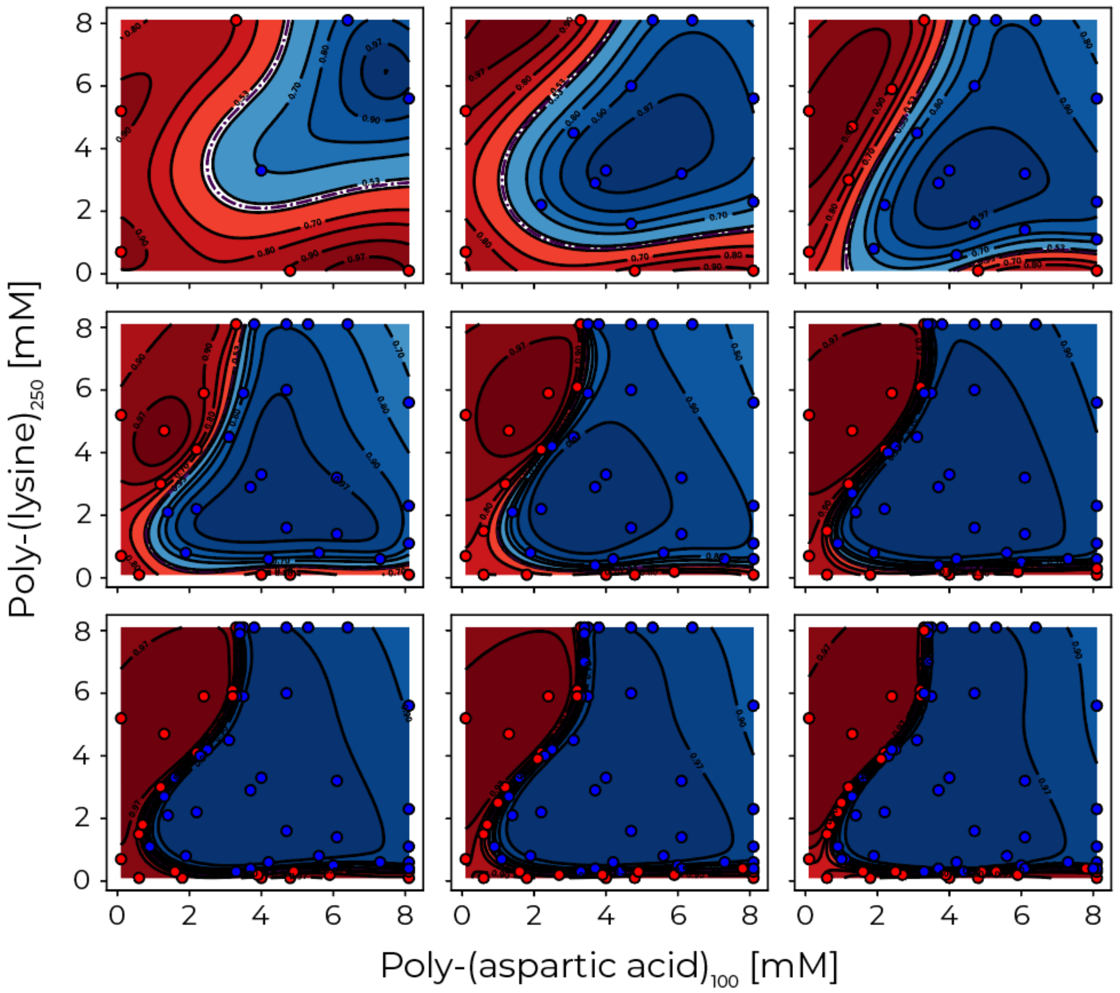

Supplementary Figure 39: Convergence of the poly-L-(lysine)$_{250}$ and poly-L-(aspartic acid)$_{100}$ condensate phase diagram. This figure shows a 72 datapoints acquired over 9 cycles. The same conditions were used as in Supplementary Figure 4 and the final phase diagram is depicted in main text Figure 5. Blue points represent phase separation, while red points indicate no phase separation. The corresponding entropy map is provided in Supplementary Figure 40. The background surface represents the model's predictions, with the scalebar provided in Supplementary Figure 3A. Axes represent monomer concentrations of each component (mM).

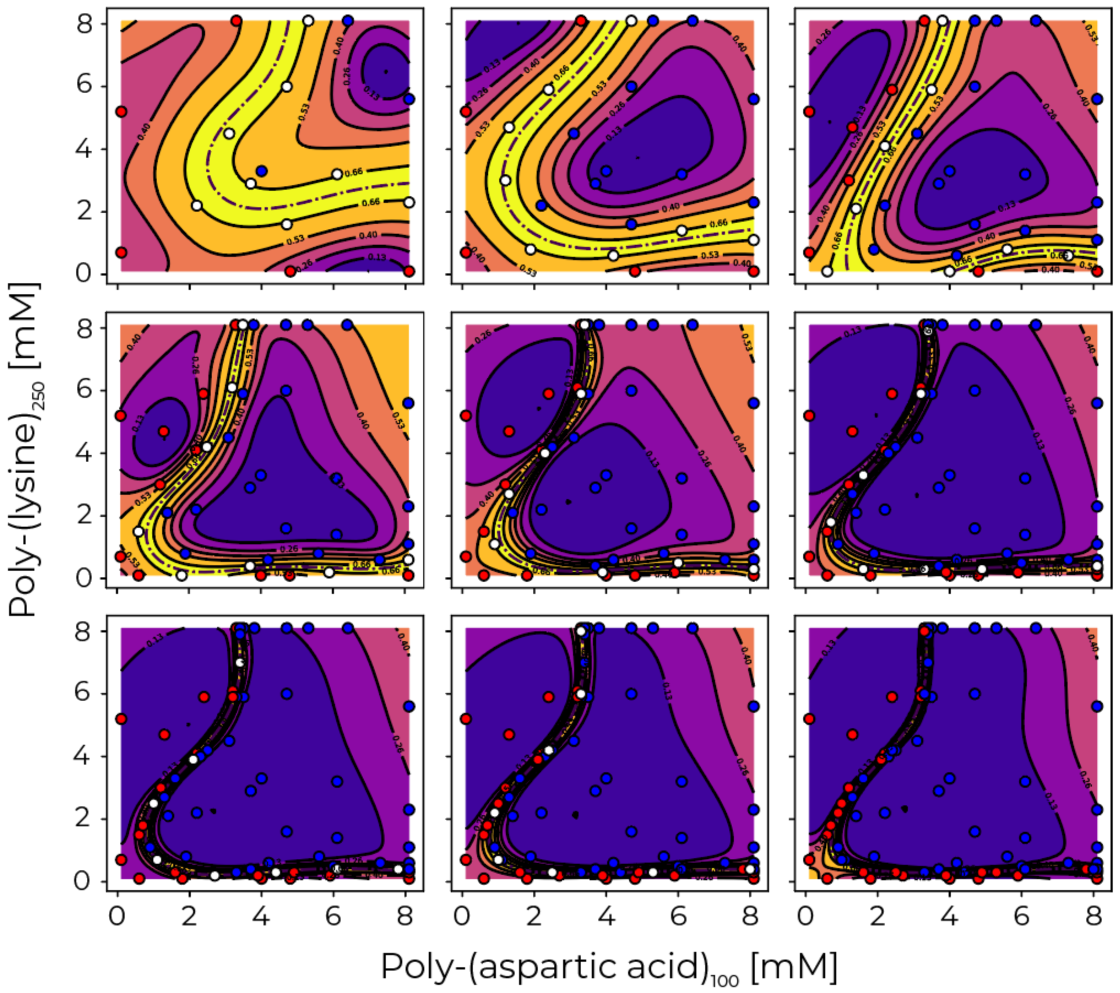

Supplementary Figure 40: Entropy map of the poly-L-(lysine)$_{250}$ and poly-L-(aspartic acid)$_{100}$ condensate phase diagram. This figure shows the entropy maps for the phase diagrams in Supplementary Figure 39. Blue points represent phase separation, red points indicate no phase separation, and white points indicate the newly requested datapoints. Axes represent monomer concentrations of each component (mM). The background surface represents the model's uncertainty, with the scalebar provided in Supplementary Figure 3B.

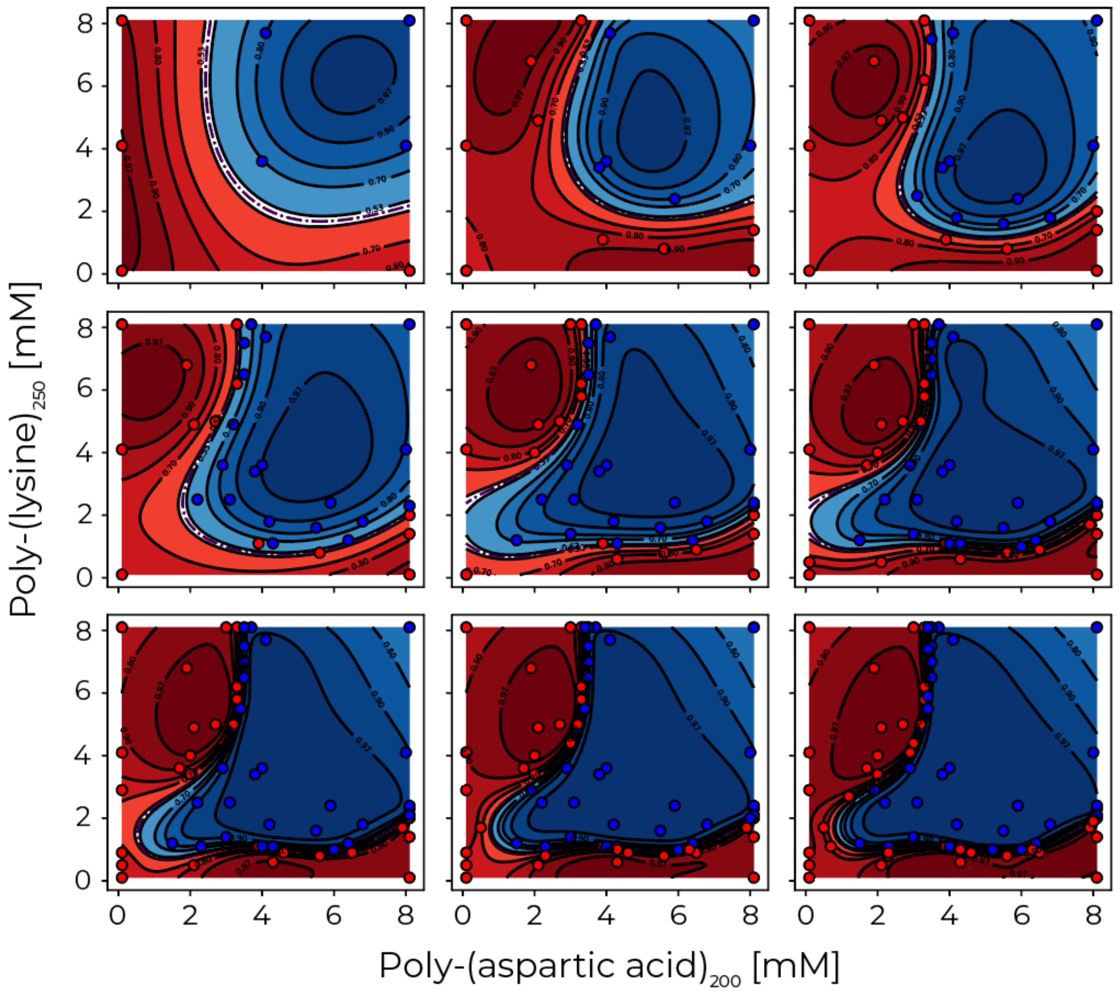

Supplementary Figure 41: Convergence of the poly-L-(lysine)$_{250}$ and poly-L-(aspartic acid)$_{200}$ condensate phase diagram. This figure shows a 72 datapoints acquired over 9 cycles. The same conditions were used as in Supplementary Figure 4 and the final phase diagram is depicted in main text Figure 5. Blue points represent phase separation, while red points indicate no phase separation. The corresponding entropy map is provided in Supplementary Figure 42. The background surface represents the model's predictions, with the scalebar provided in Supplementary Figure 3A. Axes represent monomer concentrations of each component (mM).

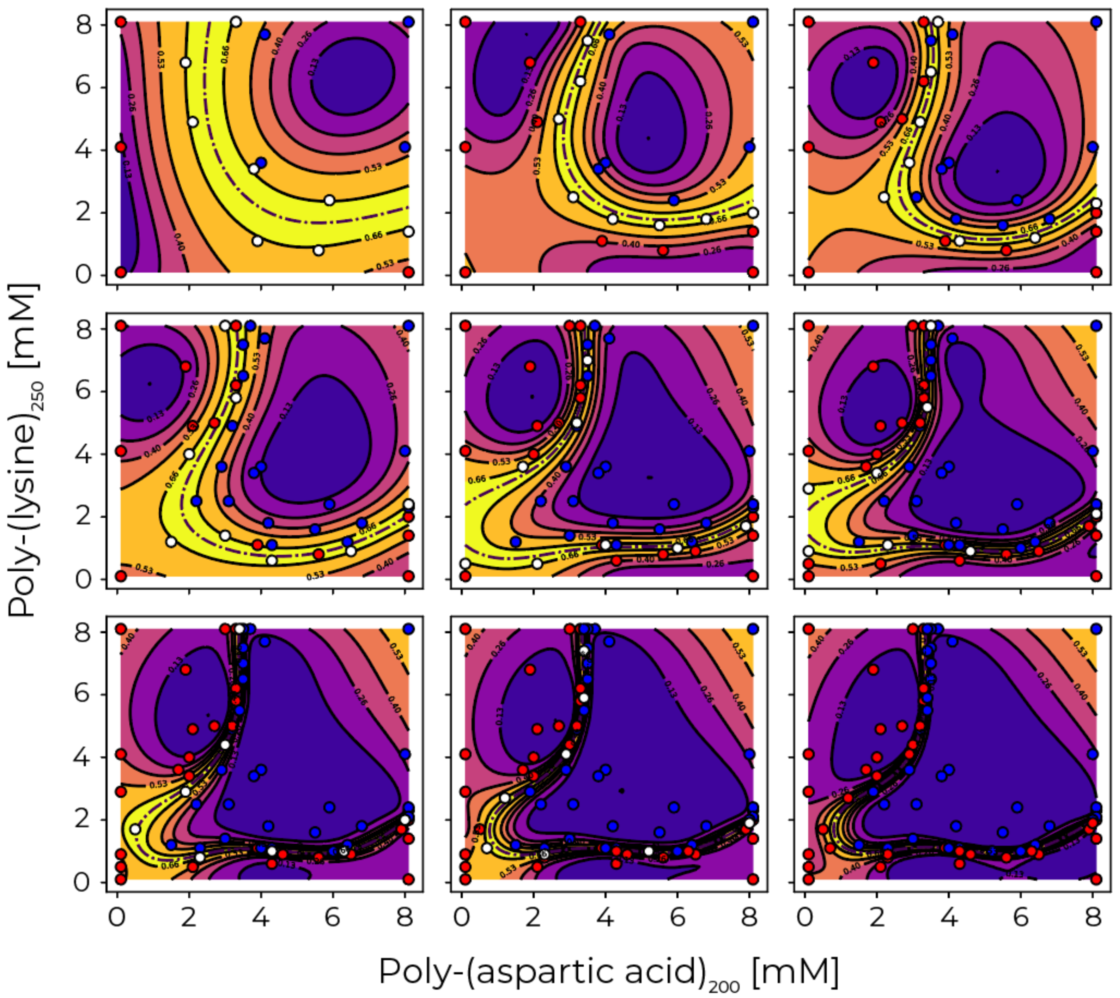

Supplementary Figure 42: Entropy map of the poly-L-(lysine)$_{250}$ and poly-L-(aspartic acid)$_{200}$ condensate phase diagram. This figure shows the entropy maps for the phase diagrams in Supplementary Figure 41. Blue points represent phase separation, red points indicate no phase separation, and white points indicate the newly requested datapoints. Axes represent monomer concentrations of each component (mM). The background surface represents the model's uncertainty, with the scalebar provided in Supplementary Figure 3B.

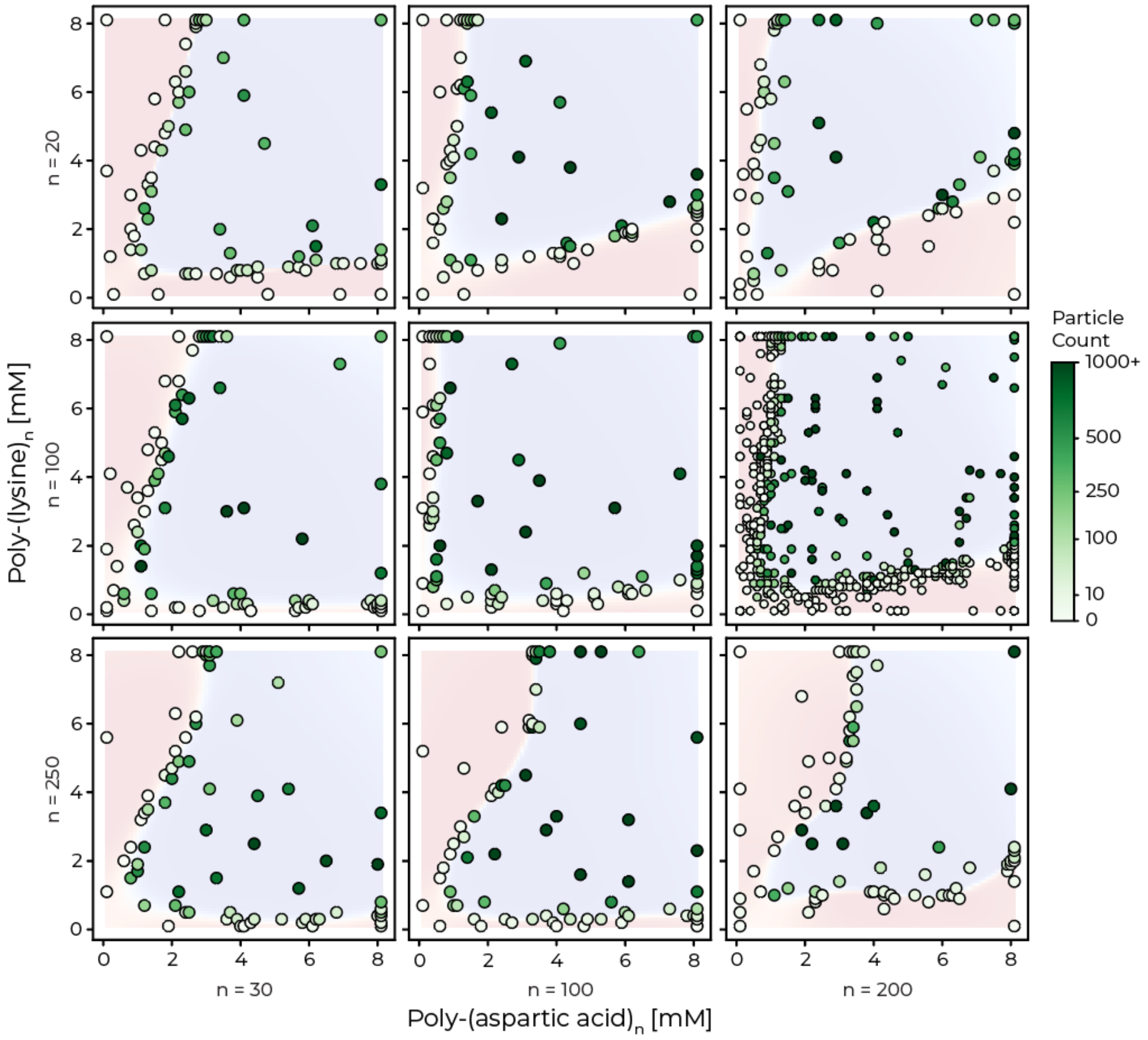

Supplementary Figure 43: Particle count mapping. This figure shows the mapping of the number of detected condensates (represented by dots) overlaid on the phase predictions in main text Figure 5. Axes represent monomer concentrations of each component (mM).

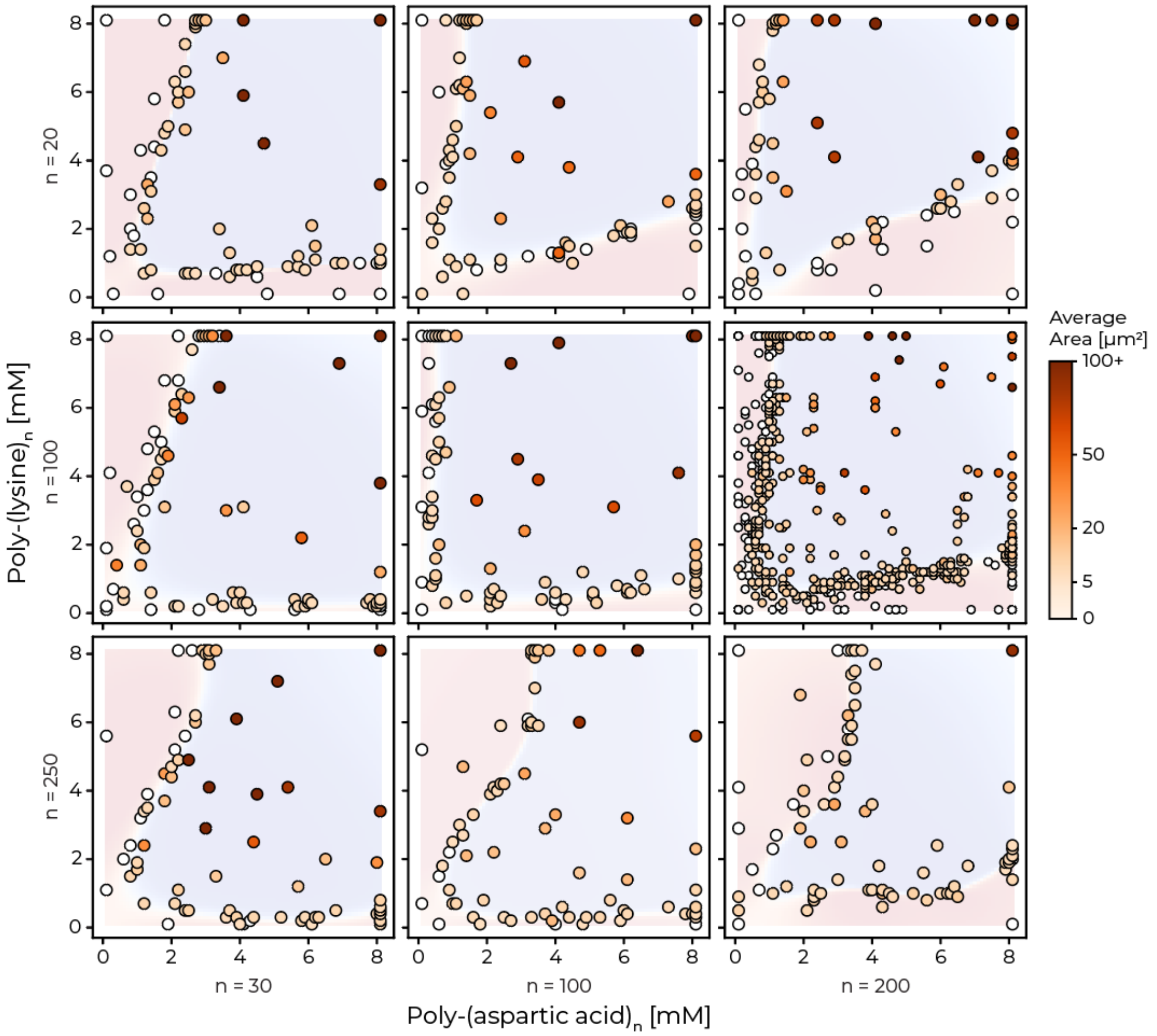

Supplementary Figure 44: Average area mapping. This figure shows the mapping of the average condensate area (represented by dots) overlaid on the phase predictions in main text Figure 5. Axes represent monomer concentrations of each component (mM).

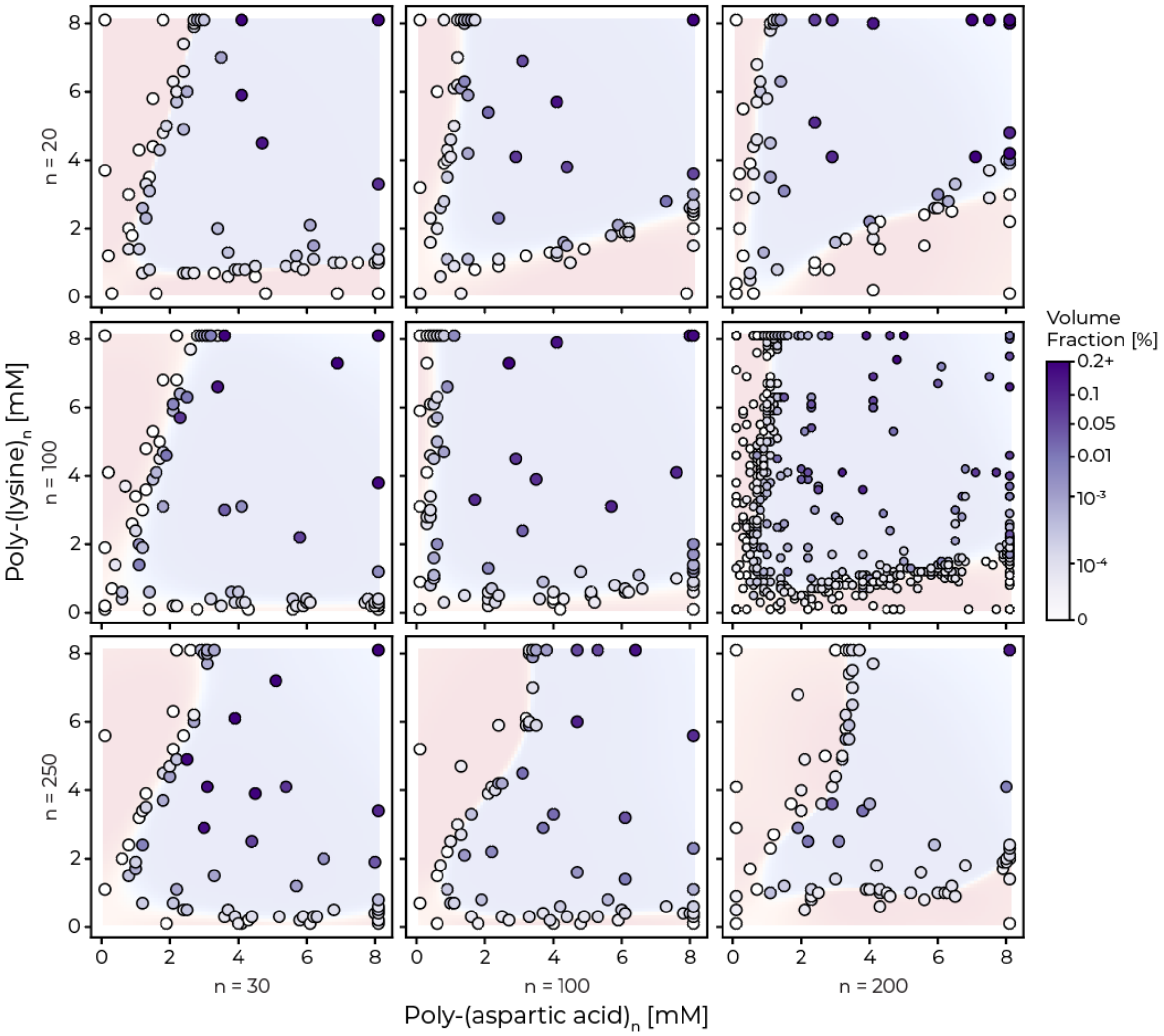

Supplementary Figure 45: Volume fraction mapping. This figure shows the mapping of condensate volume fraction (represented by dots) overlaid on the phase predictions in main text Figure 5. Axes represent monomer concentrations of each component (mM).

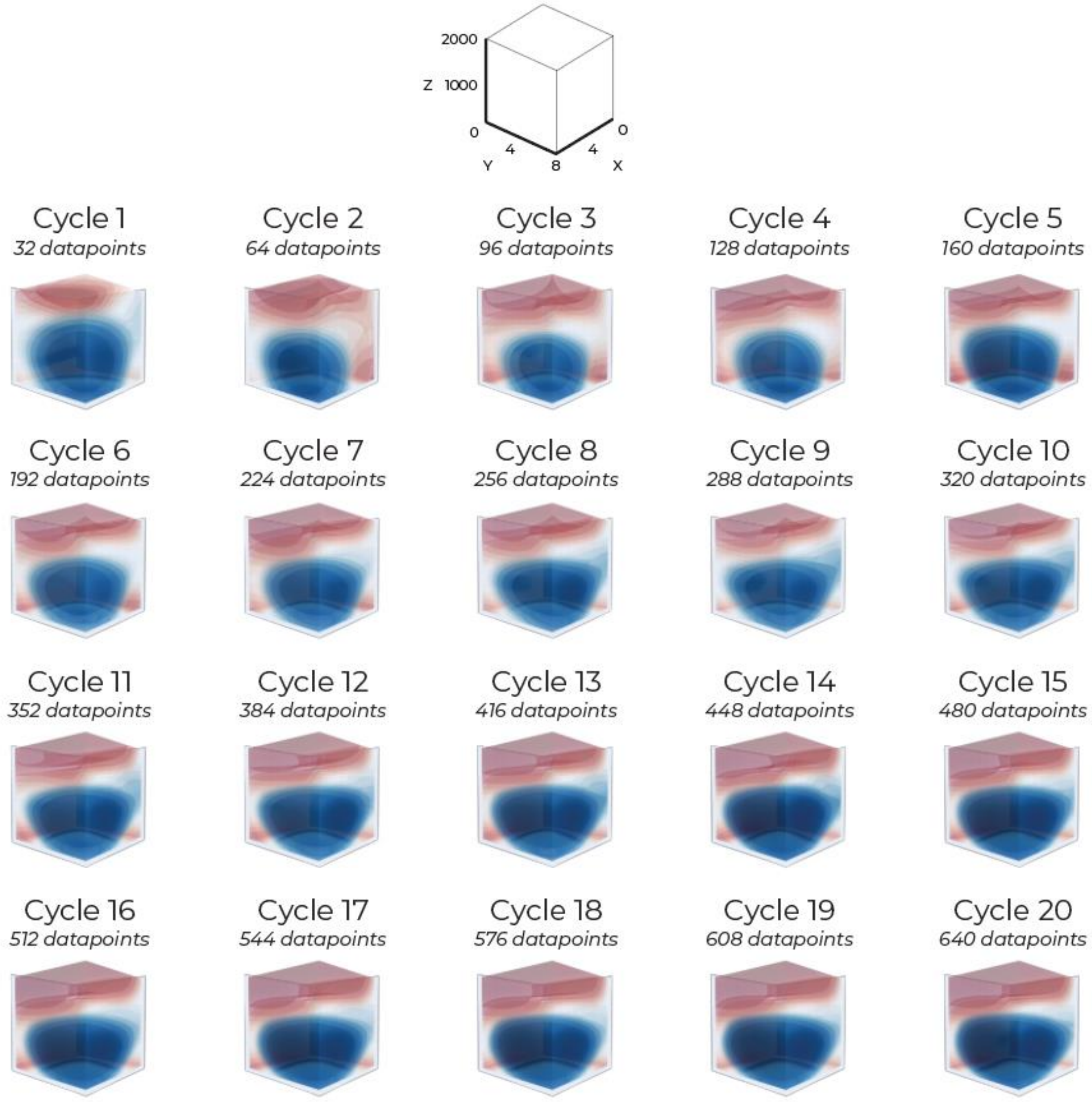

Supplementary Figure 46: Convergence of the 3D phase diagrams for poly-(lysine)$_{100}$ and poly-(aspartic acid)$_{200}$ and NaCl (Run 1). This figure shows Run 1 (of 2) where 640 datapoints were acquired over 20 cycles. Iso-probability surfaces indicate phase separation (blue, higher opacity) and no phase separation (red, lower opacity). Axes represent the salt or monomer concentrations of each component (mM). The scalebar is detailed in Supplementary Figure 3A. This overview corresponds to the data presented in main text Figure 6.

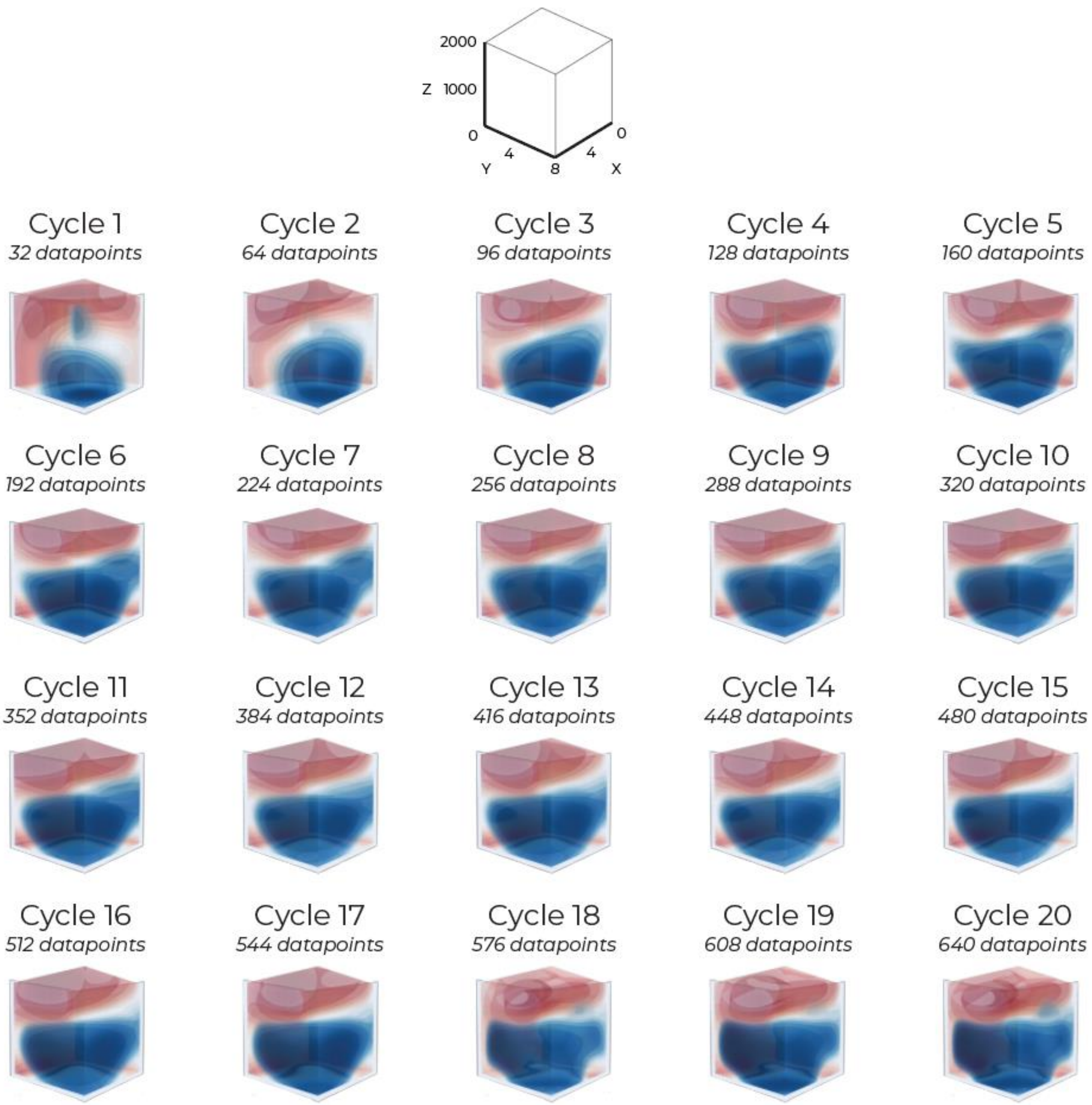

Supplementary Figure 47: Convergence of the 3D phase diagrams for poly-(lysine)$_{100}$ and poly-(aspartic acid)$_{200}$ and NaCl (Run 2). This figure shows Run 2 (of 2) where 640 datapoints were acquired over 20 cycles. Iso-probability surfaces indicate phase separation (blue, higher opacity) and no phase separation (red, lower opacity). Axes represent the salt or monomer concentrations of each component (mM). The scalebar is detailed in Supplementary Figure 3A. This overview corresponds to the data presented in main text Figure 6.

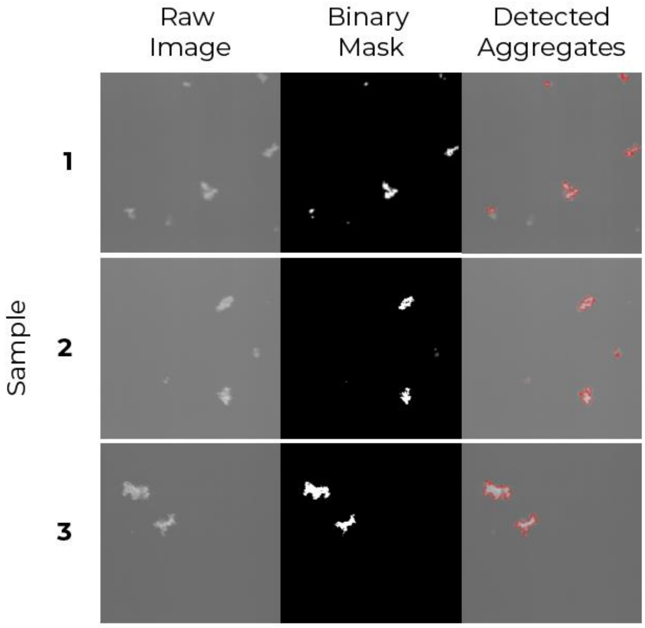

Supplementary Figure 48: Representative confocal micrographs of salt-induced aggregates formed by poly-*L*-(lysine)$_{100}$ and poly-*L*-(aspartic acid)$_{200}$ at high NaCl concentrations. From left to right: the raw confocal image, the corresponding binary mask obtained by thresholding, and an overlay highlighting detected aggregates (in red). All samples contain 0.1 mM lysine and 2050 mM NaCl, with increasing aspartic acid concentrations: 4.0, 5.7, and 7.8 mM for samples 1–3, respectively. These images illustrate the morphological appearance of the salt-induced aggregates discussed in Figure 6D of the main text.

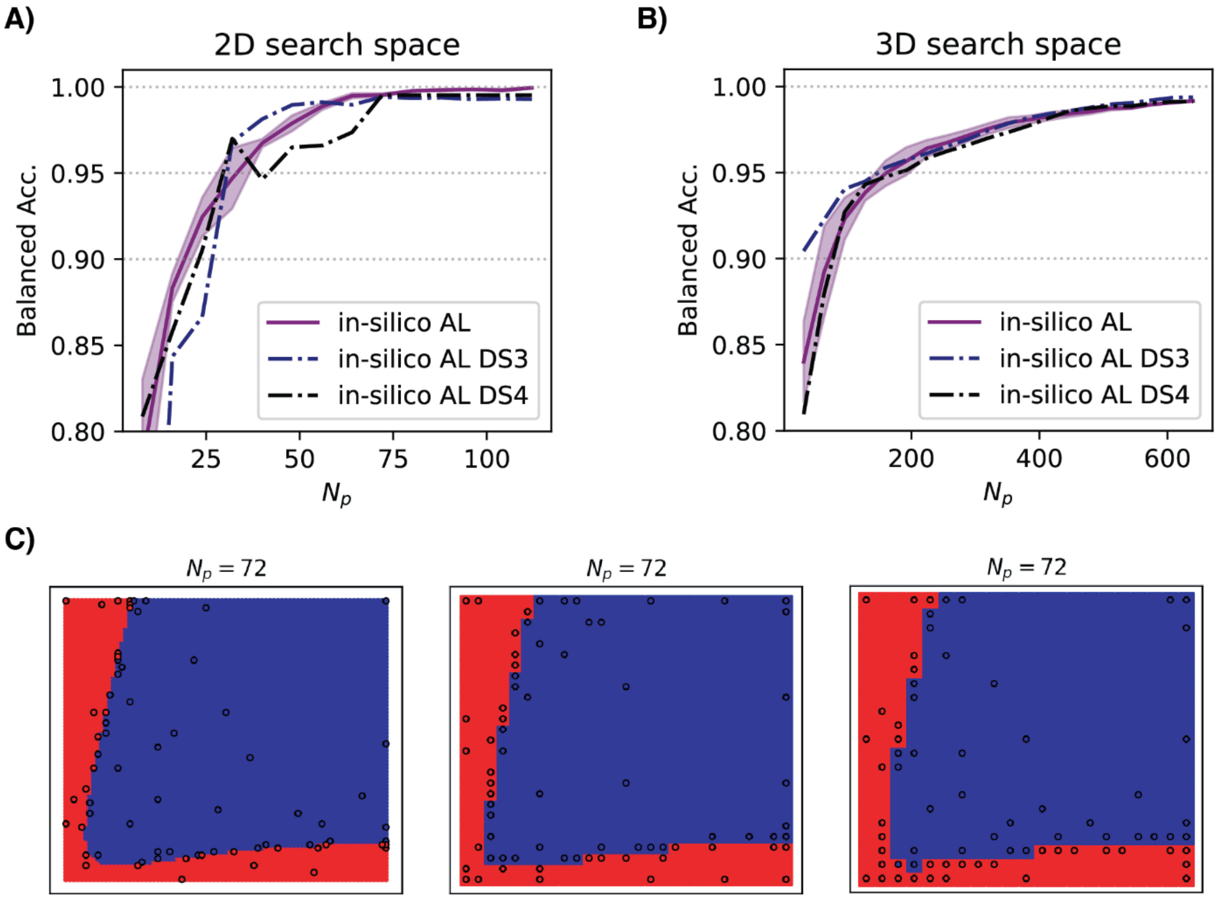

Supplementary Figure 49: Effect of search space down-sampling on in silico active learning (AL) performance. (A) Balanced accuracy per AL cycle in 2D simulations using the default search space (6561 points, solid purple line), a 3× down-sampled grid (729 points, blue dash-dotted line), and a 4× down-sampled grid (441 points, black dash-dotted line). **(B)** Balanced accuracy per AL cycle in 3D simulations using the default grid (531,441 points, solid purple line), a 3× down-sampled grid (19,683 points, blue dash-dotted line), and a 4× down-sampled grid (9261 points, black dash-dotted line). **(C)** Predicted 2D phase diagrams after 72 AL samples for the default (left), 3× down-sampled (middle), and 4× down-sampled (right) search spaces. Phase-separating conditions are shown in blue, non-separating conditions in red, and sampled points as black circles.

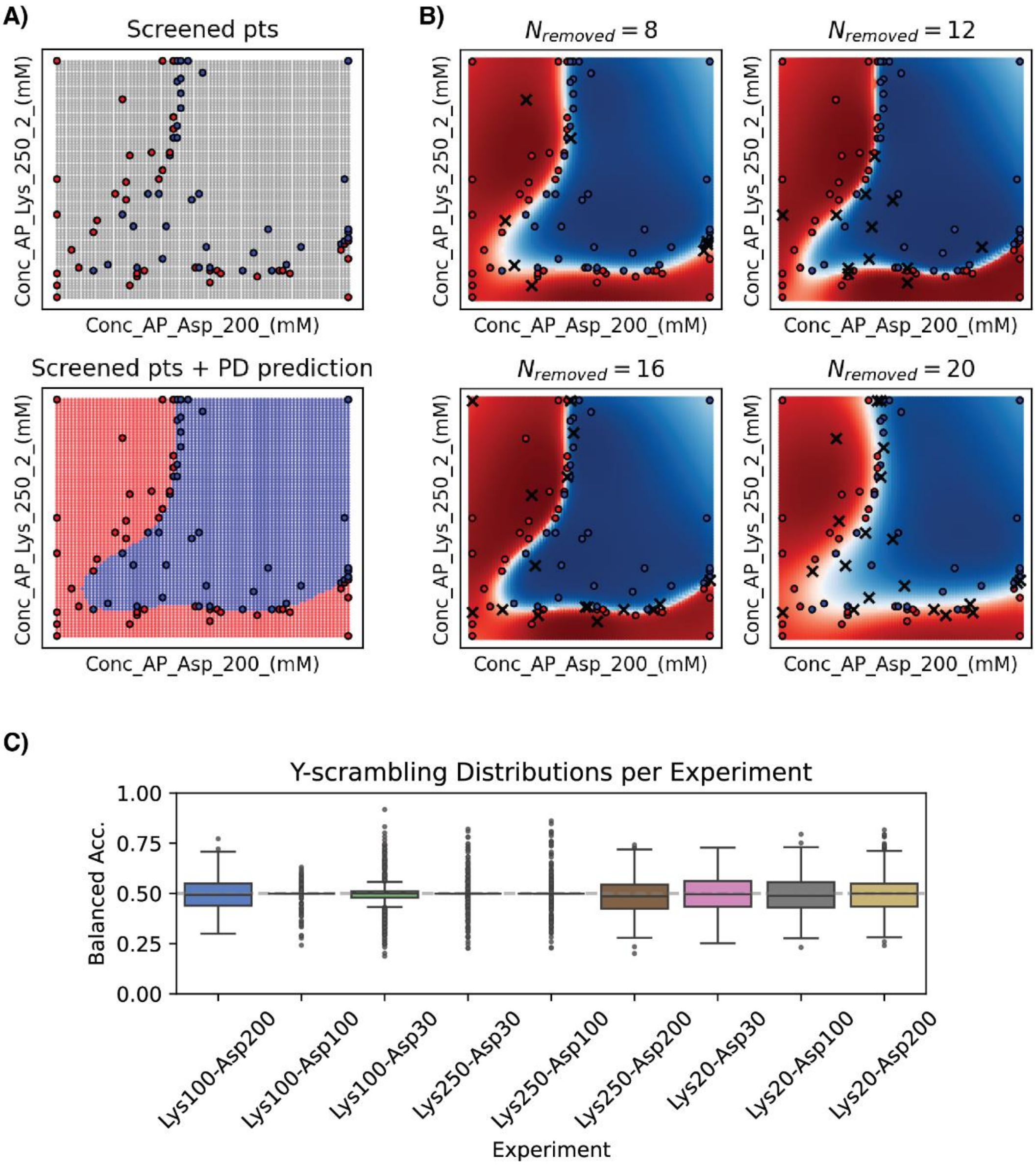

Supplementary Figure 50: Overfitting tests and analysis. A) Summary of the results for the 2D phase diagram exploration of poly-*L*-(lysine)$_{250}$ and poly-*L*-(aspartic acid)$_{200}$. The filled circles represent the screened points and are red when the recorded phase is not an aggregate or blue when the recorded phase is a coacervate. The fine grid in the background is the total 2D search space with superimposed the model prediction based on the collected experimental evidence. B) Stress-test of the model prediction by masking 8, 12, 16, or 20 of the screened points, represented by an 'X' in the figure. The masking points are chosen at random amongst the available ones. C) Y-scrambling test for each 2D experiments in main text Figure 5.
