# Supplementary material for: Automated navigation of condensate phase behavior with active machine learning
Source: Nat Commun. 2025 Oct 30;16:9598. doi: 10.1038/s41467-025-64617-2 (PMC12575655; doi:10.1038/s41467-025-64617-2)
Supplement: Supplementary file 2 — Reporting Summary [file 41467_2025_64617_MOESM2_ESM.pdf]

nature portfolio

Corresponding author(s): J.C.M. van Hest
F. Grisoni
L. Brunsveld

Last updated by author(s): Nov 28, 2024

Reporting Summary

Nature Portfolio wishes to improve the reproducibility of the work that we publish. This form provides structure for consistency and transparency in reporting. For further information on Nature Portfolio policies, see our Editorial Policies and the Editorial Policy Checklist.

Statistics

For all statistical analyses, confirm that the following items are present in the figure legend, table legend, main text, or Methods section.

n/a	Confirmed	
☐	☒	The exact sample size (*n*) for each experimental group/condition, given as a discrete number and unit of measurement
☐	☒	A statement on whether measurements were taken from distinct samples or whether the same sample was measured repeatedly
☒	☐	The statistical test(s) used AND whether they are one- or two-sided<br>*Only common tests should be described solely by name; describe more complex techniques in the Methods section.*
☒	☐	A description of all covariates tested
☒	☐	A description of any assumptions or corrections, such as tests of normality and adjustment for multiple comparisons
☐	☒	A full description of the statistical parameters including central tendency (e.g. means) or other basic estimates (e.g. regression coefficient) AND variation (e.g. standard deviation) or associated estimates of uncertainty (e.g. confidence intervals)
☒	☐	For null hypothesis testing, the test statistic (e.g. *F*, *t*, *r*) with confidence intervals, effect sizes, degrees of freedom and *P* value noted<br>*Give P values as exact values whenever suitable.*
☐	☒	For Bayesian analysis, information on the choice of priors and Markov chain Monte Carlo settings
☒	☐	For hierarchical and complex designs, identification of the appropriate level for tests and full reporting of outcomes
☒	☐	Estimates of effect sizes (e.g. Cohen's *d*, Pearson's *r*), indicating how they were calculated

Our web collection on statistics for biologists contains articles on many of the points above.

Software and code

Policy information about availability of computer code

Data collection	All components were controlled via Python (version 3.12). For data collection through confocal microscopy, pycromanager interacted with Micro-Manager (version 2.0.3) to control laser powers, Z-stacks, and XY positioning. Additionally, the Python zaber_motion library was connected to the Zaber Launcher (version 2024.11.14) to control the autofocus device.
Data analysis	All code regarding active machine learning was written in Python (version 3.12). The Python packages scikit-learn (version 1.5.0) was used for the implementation of the Gaussian Process Classifier and the calculation of the balanced accuracy. SciPy (v.1.13.1) was used for the computation of the information entropy. Pandas (v.2.2.1) was used to handle the datasets. All the other operations (e.g., design space creation, farthest point sampling, and convergence calculation) were carried out with custom scripts using NumPy (v.<2.0.0). For data visualization, matplotlib (v.3.8.4) and plotly (v.5.9.0) were used in combination with Adobe Illustrator (2024).

For manuscripts utilizing custom algorithms or software that are central to the research but not yet described in published literature, software must be made available to editors and reviewers. We strongly encourage code deposition in a community repository (e.g. GitHub). See the Nature Portfolio guidelines for submitting code & software for further information.

Data

Policy information about availability of data

All manuscripts must include a data availability statement. This statement should provide the following information, where applicable:

- Accession codes, unique identifiers, or web links for publicly available datasets
- A description of any restrictions on data availability
- For clinical datasets or third party data, please ensure that the statement adheres to our policy

The raw data generated during the active machine learning cycles, as well as the processed data used to create the manuscript's figures, are available on GitHub at https://github.com/molML/activeML-navigation-of-condensate-phases. The data at the time of publishing will be available at Zenodo at https://doi.org/10.5281/zenodo.16949697. The condensate images acquired using confocal microscopy comprise a substantial dataset and can be shared upon reasonable request to the corresponding authors.

Research involving human participants, their data, or biological material

Policy information about studies with human participants or human data. See also policy information about sex, gender (identity/presentation), and sexual orientation and race, ethnicity and racism.

Reporting on sex and gender	This study does not involve human participants, their data, or biological material; therefore, sex- and gender-based analyses are not applicable.
Reporting on race, ethnicity, or other socially relevant groupings	This study does not involve human participants, their data, or biological material; therefore, analyses based on race, ethnicity, or other socially relevant groupings are not applicable.
Population characteristics	This study does not involve human participants or biological material, so population characteristics are not relevant.
Recruitment	No participants were recruited for this study, as it does not involve human participants or biological material.
Ethics oversight	This study does not involve human participants or biological material; therefore, ethics oversight was not required.

Note that full information on the approval of the study protocol must also be provided in the manuscript.

Field-specific reporting

Please select the one below that is the best fit for your research. If you are not sure, read the appropriate sections before making your selection.

☒ Life sciences ☐ Behavioural & social sciences ☐ Ecological, evolutionary & environmental sciences

For a reference copy of the document with all sections, see nature.com/documents/nr-reporting-summary-flat.pdf

Life sciences study design

All studies must disclose on these points even when the disclosure is negative.

Sample size	Sample sizes were determined based on experimental feasibility and the number of conditions required to map phase separation behavior. No statistical methods were used to predetermine sample size.
Data exclusions	No data was excluded from the analyses.
Replication	The number of replicates for each experiment is clearly detailed in the main manuscript, most experiments were performed in duplicate or triplicate.
Randomization	Randomization was not relevant to this study as it did not involve assigning samples or participants to experimental groups. Samples were systematically prepared based on pre-defined experimental conditions.
Blinding	Blinding was not relevant to this study as it did not involve subjective assessments or group allocations that could introduce bias. All data collection and analysis steps were performed using automated methods, such as machine learning algorithms and confocal microscopy imaging.

Reporting for specific materials, systems and methods

We require information from authors about some types of materials, experimental systems and methods used in many studies. Here, indicate whether each material, system or method listed is relevant to your study. If you are not sure if a list item applies to your research, read the appropriate section before selecting a response.

Materials & experimental systems

n/a	Involved in the study
[x]	[ ] Antibodies
[x]	[ ] Eukaryotic cell lines
[x]	[ ] Palaeontology and archaeology
[x]	[ ] Animals and other organisms
[x]	[ ] Clinical data
[x]	[ ] Dual use research of concern
[x]	[ ] Plants

Methods

n/a	Involved in the study
[x]	[ ] ChIP-seq
[x]	[ ] Flow cytometry
[x]	[ ] MRI-based neuroimaging

Plants

Seed stocks	*Report on the source of all seed stocks or other plant material used. If applicable, state the seed stock centre and catalogue number. If plant specimens were collected from the field, describe the collection location, date and sampling procedures.*
Novel plant genotypes	*Describe the methods by which all novel plant genotypes were produced. This includes those generated by transgenic approaches, gene editing, chemical/radiation-based mutagenesis and hybridization. For transgenic lines, describe the transformation method, the number of independent lines analyzed and the generation upon which experiments were performed. For gene-edited lines, describe the editor used, the endogenous sequence targeted for editing, the targeting guide RNA sequence (if applicable) and how the editor was applied.*
Authentication	*Describe any authentication procedures for each seed stock used or novel genotype generated. Describe any experiments used to assess the effect of a mutation and, where applicable, how potential secondary effects (e.g. second site T-DNA insertions, mosiacism, off-target gene editing) were examined.*
